# Deep Immune Phenotyping and Single-Cell Transcriptomics Allow Identification of Circulating TRM-Like Cells Which Correlate With Liver-Stage Immunity and Vaccine-Induced Protection From Malaria

**DOI:** 10.3389/fimmu.2022.795463

**Published:** 2022-02-07

**Authors:** Andrés Noé, Mehreen S. Datoo, Amy Flaxman, Mohammad Ali Husainy, Daniel Jenkin, Duncan Bellamy, Rebecca A. Makinson, Richard Morter, Fernando Ramos Lopez, Jonathan Sheridan, Dimitrios Voukantsis, Naveen Prasad, Adrian V. S. Hill, Katie J. Ewer, Alexandra J. Spencer

**Affiliations:** ^1^ The Jenner Institute, University of Oxford, Oxford, United Kingdom; ^2^ Department of Radiology, John Radcliffe Hospital, Oxford, United Kingdom; ^3^ Bioinformatics Hub, Department of Oncology, University of Oxford, Oxford, United Kingdom

**Keywords:** malaria vaccine, malaria vaccine decelopment, correlates of protection (CoP), TRM, tissue resident memory CD8+ T cells, tissue resident memory T cell, scRNA seq

## Abstract

Protection from liver-stage malaria requires high numbers of CD8+ T cells to find and kill *Plasmodium*-infected cells. A new malaria vaccine strategy, prime-target vaccination, involves sequential viral-vectored vaccination by intramuscular and intravenous routes to target cellular immunity to the liver. Liver tissue-resident memory (TRM) CD8+ T cells have been shown to be necessary and sufficient for protection against rodent malaria by this vaccine regimen. Ultimately, to most faithfully assess immunotherapeutic responses by these local, specialised, hepatic T cells, periodic liver sampling is necessary, however this is not feasible at large scales in human trials. Here, as part of a phase I/II *P. falciparum* challenge study of prime-target vaccination, we performed deep immune phenotyping, single-cell RNA-sequencing and kinetics of hepatic fine needle aspirates and peripheral blood samples to study liver CD8+ TRM cells and circulating counterparts. We found that while these peripheral ‘TRM-like’ cells differed to TRM cells in terms of previously described characteristics, they are similar phenotypically and indistinguishable in terms of key T cell residency transcriptional signatures. By exploring the heterogeneity among liver CD8+ TRM cells at single cell resolution we found two main subpopulations that each share expression profiles with blood T cells. Lastly, our work points towards the potential for using TRM−like cells as a correlate of protection by liver-stage malaria vaccines and, in particular, those adopting a prime-target approach. A simple and reproducible correlate of protection would be particularly valuable in trials of liver-stage malaria vaccines as they progress to phase III, large-scale testing in African infants. We provide a blueprint for understanding and monitoring liver TRM cells induced by a prime-target malaria vaccine approach.

## Introduction

Malaria is the most problematic parasitic disease in human history. A highly efficacious vaccine could curb the hundreds of millions of cases and hundreds of thousands of deaths occurring each year. It has been over 30 years since Ruth and Victor Nussenzweig identified that protection from liver-stage malaria requires high numbers of CD8+ T cells to find and kill *Plasmodium*-infected cells (1). To this end, substantial effort has been invested in optimising viral vector strategies able to generate high frequencies of antigen-specific CD8+ T cells (2–7). A prominent approach entails vaccinating with heterologous viral vectors to induce CD8+ T cells and results in modest clinical efficacy in both malaria naïve and pre-exposed individuals (4, 8). CD8+ T cell numbers following vaccination correlate with efficacy, suggesting that increased numbers of circulating CD8+ T cells are associated with improved protection.

A new malaria vaccine strategy, prime-target vaccination (PTV), involves sequential viral-vectored vaccination by intramuscular and intravenous routes to target cellular immunity to the liver (9). The efficacy of leading liver-stage malaria vaccine candidates in mice can be enhanced with this approach from 0-30% to 100% efficacy. PTV substantially increases the number of antigen-specific tissue-resident memory (TRM) CD8+ T cells in the livers of mice (9). This and other studies have shown that this specialised subset of hepatic T cells can be induced by heterologous prime-boost vaccines and that they are necessary and sufficient for protection against liver-stage rodent malaria (9–11).

Relatively little is known about the human liver T cell population in health. It is now well established that TRM cells represent a functionally distinct compartment poised at portals of pathogen entry to provide local protection (12–15). Short of showing long-term residence in tissues and/or lack of recirculation, TRM cells can be identified by using a number of markers found on their cell surface. Identifying surface markers of human liver residency is an ongoing endeavour; CD69, CXCR3 and, to a lesser extent, CD103 are often used to distinguish TRM cells from other hepatic T cells (12, 16–18). PD-1, a marker of T cell hyporesponsiveness (19), and CXCR6, a chemokine that can mediate lymphocyte recruitment (20), are two other important markers of hepatic TRM cells (12, 21). Several reports have described core transcriptional signatures for TRM cells from multiple tissues identified by bulk (14), and single-cell RNA-sequencing (22–24).

The challenges associated with mapping hepatic immunological populations are threefold: i) access-related, ii) rare populations are thought to be critical mediators of immunity (such as TRM cells), and iii) highly specialised techniques are often required (ie. in sample collection and preparation, and analyses). The added complexity of trying to understand hepatic immunity in the context of malaria vaccine-induced immunity, at scale, makes for a seemingly insurmountable task. It is therefore critical to identify if inferences about the liver can be made by looking at the blood; that is, to identify correlates of hepatic immunity and of protection. Recently there have been reports that TRM cells from various sites can exit tissues in response to stimuli, re-enter the circulation and even contribute to the expansion of memory T cells (25–28). Indeed, small but significant proportions of circulating T cells have been demonstrated to express ‘tissue-resident signature’ genes at levels comparable to T cells in tissues (23). Therefore, we wanted to investigate the qualities of human hepatic TRM cells and determine whether there was a comparable or related subset of T cells in the blood.

Herein, we track the phenotype, transcriptomics and kinetics of blood-derived TRM-like cells with the aim of identifying a correlate of malaria vaccine-induced immunity against controlled human malaria infection (CHMI). A detailed understanding of TRM cells and any potential circulating counterpart in humans, as well as an appreciation of their relationship, will likely be critical in identifying correlates of liver-stage malaria protection and further vaccine development.

## Materials and Methods

### Volunteers

As part of a phase I/IIa sporozoite challenge study to assess intravenous boosting (prime-target) with malaria vaccine candidates ChAd63 and MVA encoding ME−TRAP (**Supplementary Figure 1**, ClinicalTrials.gov Identifier: NCT03707353, Datoo et al. in preparation), T cells from the liver and blood were isolated and compared. An aim of this comparison was to investigate a translatable liver-stage malaria correlate of immunity, by using insights gained from studying liver TRM cells. All recruited volunteers were healthy, malaria naïve adults aged between 18 and 45 years with PBMCs isolated at multiple timepoints throughout the study. Fifteen of the thirty-nine participants were asked to participate in liver fine needle aspirate (FNA) sampling at a single timepoint after intravenous vaccine administration, with volunteers recruited equally across the four groups. The study protocol and associated documents were reviewed and approved by the UK National Research Ethics Service, Committee South Central–Oxford REC A (18/SC/0384) and the Medicines and Healthcare Products Regulatory Agency (EudraCT: 2017-001075-23). CHMI (“challenge”) after vaccination and diagnosis of malaria was conducted as we previously described (5).

### Sample Collection and Cell Isolation

Liver FNA was performed as previously described (29), with a few modifications. Briefly, under ultrasound guidance a 22-gauge spinal needle with an internal trocar was used to gain access, along an anaesthetised tract, to the edge of the liver capsule. The internal trocar was removed after a further 2-3cm insertion, into the parenchyma. Liver cells were collected into a 20mL syringe containing 3mL of cold sterile saline. Aspiration was performed as the needle was withdrawn 1-2cm, but while still remaining in the liver parenchyma using a ‘fanning technique’ (30, 31). Cells were transferred into a fresh 50mL tube containing 30ml catch media (RPMI supplemented with 25mM HEPES and 15IU/mL heparin) by flushing the syringe and needle with 3mL of media. Two aspirates per volunteer were performed prior to transfer on ice for immediate processing. Any aspirate with frank blood were discarded.

Blood samples for PBMC separation were collected in heparinised tubes and separated on a centrifugation gradient using Lymphoprep (Axis Shield) within 4 hours of venepuncture. Liver samples were resuspended in RBC lysis solution (eBioscience) for less than five minutes, before counting and staining. FNA and PBMC sampling was performed 16-24 days after IV viral vector administration.

### Flow Cytometry and Intracellular Cytokine Staining

For the characterisation of T cell kinetics after IV viral vector, freshly isolated PBMC were stimulated with anti-CD28 and anti-CD49d at 1μg/mL (Becton Dickinson), 200μg/mL of CD107a-PeCy5 (eBioscience) together with a pool of all 56 peptides of the T9/96 strain *P. falciparum* TRAP antigen (final concentration 5µg/mL) for 16-20 hours (32), 5μg/ml Staphylococcal enterotoxin B (SEB) (Sigma Aldrich) or media (unstimulated). Brefeldin A and monensin (eBioscience), both at 1 μg/mL, were added after two hours. For lymphocytes used in matched PBMC and FNA characterisation, no peptide stimulation was performed.

Following surface staining, fixation and intracellular staining (see **Supplementary Materials and Methods** for antibody list), acquisition was performed using an LSRII or LSRFortessa X-20 SORP (BD Biosciences). At least 500,000 events were acquired per sample, with data analysed on FlowJo version 10 (BD Biosciences). Lymphocytes were gated on live, size, and singlet (FSC-A vs FSC-H and FSC-A vs FSC-W) and dead cells (Live/Dead amine reactive+), monocytes (CD14+) and B cells (CD19+) were excluded. Cells were subsequently gated on CD45+ CD3+ CD8+ T cells.

### RNA Sequencing

Cell sorting was conducted using a FACSAriaIII (BD Biosciences) using a 70μm nozzle and single cell purity. All mini-bulk samples consisted of 100 cells and were attained using a two-way sort based on CD69 status (positive or negative), pre-gated on live single CD20- CD45+ CD3+ CD4- CD8+ CD45RA- cells. All single cells were CD69+. Mini-bulk samples were collected into 0.2mL PCR tubes and single cells directly into RNAse free 96-well PCR plates with 4.01µL lysis catch buffer (0.4% (vol/vol) Triton X-100 and 2U/μl RNase inhibitor, 4x10^7^ dilution of ERCC spike-in RNA control, 2.5mM dNTPs (Thermo-Fisher), 2.5µM Oligo-dT30VN). Samples were vortexed, spun and placed on dry ice within 10 minutes. Smart-Seq2 libraries were generated following the established protocol (33).

### Statistical and Bioinformatic Analyses

Prism version 8 (GraphPad) and/or RStudio (base R version 3.6.2) were used for analyses. Mean values with standard deviation are shown in all graphs, unless otherwise stated. Significance testing of differences between group means (for normally distributed data) used the two-tailed Student’s t-test, or medians used the two-tailed Mann–Whitney U-test. Univariate immunological analysis compared time to malaria diagnosis between strata dichotomised by volunteer T cell proportions. Multiple regression Cox proportional hazards models were fitted to flow cytometry data with frequency of T cell populations as the independent variable and time to diagnosis as the dependent variable. Models were assessed as previously described (4); Akaike’s information criterion (AIC) was used as an aid for choosing between competing nested models. Lower AIC values indicate a preferred model that maximises model parsimony. Immunological correlations with time to patent parasitaemia or other variables were pre-specified and prioritised analysis of T cell subsets based on observations from pre-clinical studies using prime-target vaccination. An alpha level of 0.05 was considered statistically significant.

Sequencing reads were aligned to the human genome using STAR (34). Ensembl gene counts were generated using featureCounts (35). DESeq2 v3.10 (36) was used for normalisation and feature selection in analysis of the mini-bulk experiment. The Wald test was the default used for hypothesis testing. Seurat (37, 38) was used for normalisation, variance stabilisation and feature selection in the single cell experiment. Differential expression was based on the non-parameteric Wilcoxon rank sum test (see **Supplementary Materials and Methods** for further information).

## Results

### Hepatic T Cells Correlate Quantitatively to Matched Peripheral Blood T Cells

**Figure 1:**
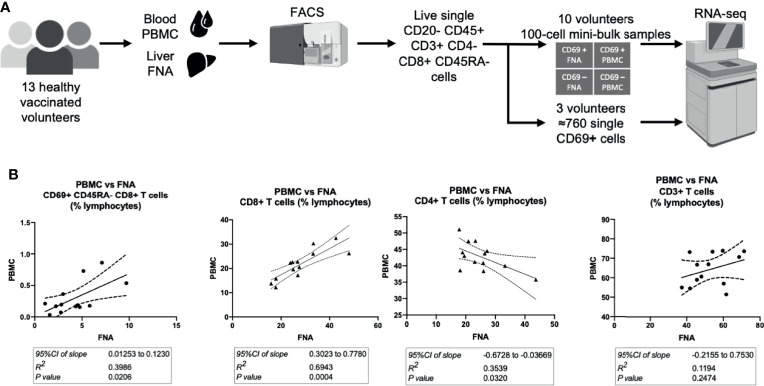
Hepatic and circulating lymphocytes correlate numerically and phenotypically when analysed by flow cytometry. **(A)** Sampling workflow. 15 volunteers across all vaccination groups were recruited for liver FNA sampling. Two volunteers were unable to provide samples, due to logistical reasons, and withdrew on the sampling days. Hepatic fine needle aspirate and peripheral venesection were performed within one hour. Lymphocytes were isolated, stained for flow cytometry/cell sorting and sorted within three hours of sampling. Ten volunteers’ FNA and PBMC samples were two-way sorted for 100-cell mini-bulk RNA-sequencing and three volunteer samples were sorted for single-cell RNA-sequencing. **(B)** Quantitative correlations between liver and blood samples. The 95%CI of linear regression slope, R2 value of goodness of fit and the p value that the slope is significantly non-zero are presented below the respective plots. The P values were calculated by F tests. All plots are derived from 13 matched FNA and PBMC samples. All values are presented as a proportion of single CD45+ lymphocytes in each sample. FNA, fine needle aspirate. PBMC, peripheral blood mononuclear cells. tSNE, t-distributed stochastic neighbour embedding. CI, confidence interval. FNA, fine needle aspirate. GMFI, geometric mean fluorescence intensity. PBMC, peripheral blood mononuclear cells. tSNE, t-distributed stochastic neighbour embedding.

Following intravenous vaccination with a malaria vaccine candidate, the frequency and phenotype of TRM cells isolated from the liver by FNA sampling and peripheral blood were compared (**Figure 1A**). Using CD69 and CD11a positivity to identify TRM cells in the liver we observed an enrichment of these CD8+ T cells in the liver (**Table 1** and **Supplementary Figures 2**, **3**), consistent with previous reports (12, 30, 39). In contrast, memory CD8+ T cells expressing CD103 did not differentially populate the liver or blood (**Table 1**). Using an algorithm (30, 39) developed to determine the “liver−like” score of an FNA sample, showed that our sample quality was comparable with and, indeed, more representative of the liver (median liver-like score 78%, IQR 23.6%) than previously reported (39).

**Table 1:** Cells expressing TRM cell markers.

Population	FNA	PBMC	p value
**CD8+ (%CD8+ T cells)**	*Median (IQR)*	*Median (IQR)*	
* CD69+ CD45RA-*	13.80 (12.27)	0.380 (0.84)	0.0002
* CD69+ CD11a hi CD45RA-*	13.70 (12.26)	0.365 (0.83)	0.0002
* CD103+ CD45RA-*	1.280 (1.00)	1.29 (0.94)	0.4143
* CD103+ CD11a hi CD45RA-*	1.090 (0.87)	0.950 (0.75)	0.4143
* CD69+ CD103+ CD11a hi CD45RA-*	0.5200 (0.62)	0.00516 (0.01)	0.0002
**CD4+ (%CD4+ T cells)**			
* CD69+ CD45RA-*	4.55 (4.01)	0.15 (0.12)	0.0002
* CD69+ CD11a hi CD45RA-*	4.31 (4.03)	0.14 (0.12)	0.0002
* CD103+ CD45RA-*	ND	ND	NA
* CD103+ CD11a hi CD45RA-*	ND	ND	NA
* CD69+ CD103+ CD11a hi CD45RA-*	ND	ND	NA

Populations of CD8+ and CD4+ T cells expressing TRM cell markers. Representative flow cytometry plots from FNA and PBMC of the same donors is shown in **Supplementary Figure 2**. The gating strategy for each of these populations is shown in **Supplementary Figure 3**. Wilcoxon matched pairs signed rank tests were used to check for significance of differences between FNA and PBMC samples. An exact p value is reported. Values reported are medians with interquartile ranges. Effective pairing, checked by Spearman correlation coefficient, was present for all samples (p<0.05). FNA, fine needle aspirate. IQR, interquartile range. NA, not applicable. ND, not detected. PBMC, peripheral blood mononuclear cells. TRM, tissue-resident memory T cells.

Cells with a TRM cell phenotype (ie. CD69^+^ memory T cells) in the liver correlated significantly to a similar population observed in the blood (**Figure 1B**), present at a much lower frequency. The frequency of total CD8+ CD3+ CD56- T cells significantly positively correlated between PBMC and FNA samples. PBMC CD4+ CD3+ CD56- T cell frequency negatively correlated to matched FNA T cell frequencies (**Figure 1B**), but this was driven by one outlying volunteer. Consistent with previous reports, a higher frequency of both TRM cells and CD8+ T cells were observed in the liver compared to blood (39).

Phenotypic characterisation of FNA and PBMC samples showed subsets of cells expressing TRM cell functional molecules. Regardless of tissue, CD69+ memory CD8+ T cells had on average a higher frequency of CXCR6+, CD103+, and PD−1+ cells and higher level of marker expression compared to CD8+ CD69- counterparts (**Supplementary Figure 4**). This was not evident in the CD103+ CD69+ cell proportion in PBMC, however, suggesting CD103 may be differentially expressed between liver and blood CD69+ memory T cells (**Supplementary Figure 4**, left panes). Using a t-distributed stochastic neighbour embedding algorithm to visualise and examine the co-expression of TRM cell markers on CD8+ memory T cells (**Supplementary Figure 5**), we identified three independent clusters, with little or no overlap between CD69+ and CD69- CD45RA- cells (**Supplementary Figures 5B, C**), signifying their relative enrichment in the CD69+ compartment. Differential expression of CXCR6, PD−1 and CD103 were the main drivers of cluster formation, with no substantial differences in the expression patterns of other markers (**Supplementary Figure 5D**). All three clusters co-occurred in FNA and PBMC samples, however Subset 2 appeared at a lower frequency in PBMC samples compared to FNA samples. The presence of circulating cells with established markers of tissue residency or ‘circulating TRM cells’ goes against the traditional definition of resident cells. We use “TRM-like cells” herein, instead of “circulating TRM cells”, to indicate the (PBMC) circulating population of T cells that share phenotypic characteristics with bona fide (FNA) liver TRM cells.

### The CD8+ Memory T Cell Transcriptome Can be Defined by CD69 Status

**Figure 2:**
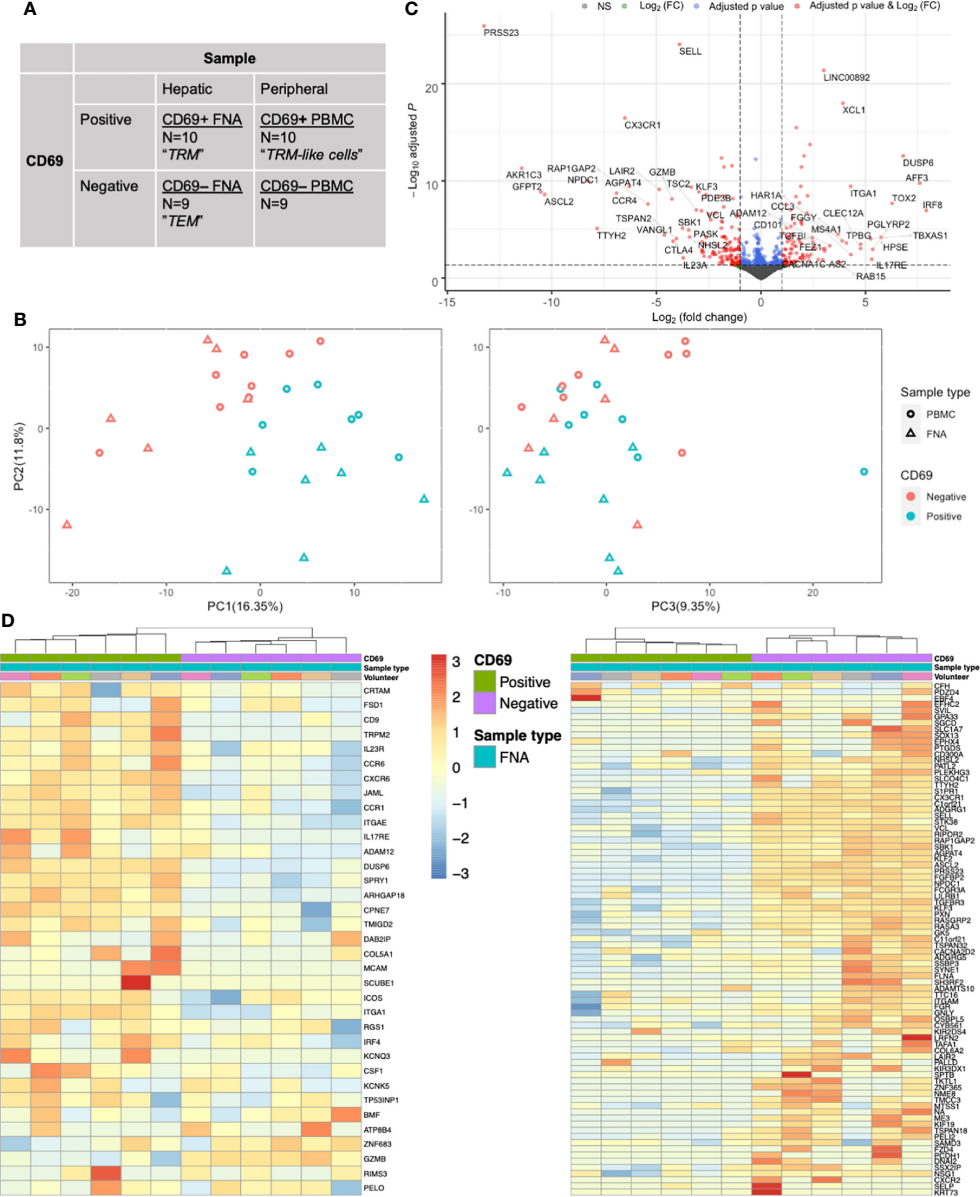
CD69 status is a major driver of transcriptional differences in memory CD8+ T cells as assessed by mini-bulk RNA-seq. **(A)** Mini-bulk RNA-seq experimental design. Each category was composed of N number of 100-cell samples pre-gated on live single CD20- CD45+ CD3+ CD4- CD8+ CD45RA- cells sequenced in bulk by the SmartSeq2 protocol. Two volunteers’ samples and one FNA paired (CD69+ and CD69-) samples were removed during QC. Non-normalised counts were input to DESeq2 and samples were paired according to volunteer and sample type. Differential expression analyses used a generalised linear model where counts were modelled using a negative binomial distribution. The Wald test was the default used for hypothesis testing when comparing gene expression between two sets of paired variables. See **Figure 1A** for sampling workflow. **(B)** PCA plots based on the 500 most variable genes by mini-bulk RNA sequencing. Plots of the first three PC, coloured according to CD69 status and sample type of the sequenced cells. Separation of CD69+ and CD69- cells was a composite of PC1 and PC2. PC3 does not distinguish CD69 status. Twenty-eight samples were analysed. **(C)** Volcano plot of up- and down-regulated genes between paired FNA CD69+ samples and CD69- samples. The top 50 up- and down-regulated genes have been annotated. Positive log(FC) values indicate greater gene expression in CD69+ samples, compared to CD69- samples. Negative log(FC) values indicate greater gene expression in CD69- samples. Differential expression of the 12,515 genes was tested for significance using the Wald test. P values were adjusted using the Benjamini-Hochberg (false discovery rate) correction. **(D)** Gene heatmap of FNA samples using the core TRM cell transcriptional signature described by Kumar et al. Left, heatmap of genes that are upregulated in the CD8+ Kumar et al. core transcriptional signature. Right, heatmap of genes that are downregulated in the CD8+ Kumar et al. core transcriptional signature. Columns are clustered based on Spearman’s correlation of rlog normalised count values. Rows are clustered according to Pearson correlation of the rlog normalised gene counts. Gene count values are centred and scaled across rows. FACS, fluorescence-assisted cell sorting; FC, fold change; FNA, fine needle aspirate; PBMC, peripheral blood mononuclear cells; PC, principal component; PCA, principal component analysis; QC, quality control; TEM, effector memory T cell; TRM, tissue-resident memory T cell.

To determine whether surface expression of CD69 would also define TRM and TRM-like cells at the transcriptome level, we performed mini-bulk RNA sequencing on both CD69 positive and negative cells isolated from liver FNA and matched PBMC samples (**Figures 1A**, **2A**). CD69 status accounted for much of the variability found in the mini-bulk samples (**Figure 2B**). Differential gene expression between liver CD69+ and CD69- cells was consistent with previous resports (**Supplementary Data File 1**). The top 50 up-regulated genes included *IRF8*, *TOX2* and *CCL3* and down-regulated genes included *CCR4*, *IL23A* and *TSC2* (**Figure 2C**), as well as a large proportion of genes known to be important in T cell residency (14, 22, 40). Using a CD8+ TRM cell transcriptional signature generated by Kumar and colleagues (14), hierarchical clustering differentiated TRM and effector memory T (TEM) cells into independent populations (**Figure 2D**). Thirteen of the 25 most down-regulated genes and five of the 25 most up-regulated genes, between FNA CD69+ and CD69- cells, were present in the Kumar et al. signature. Gene set enrichment analysis (GSEA) of the genes upregulated in the Kumar et al. signature showed significant normalised enrichment in CD69+ cells from both FNA and PBMC comparisons (**Supplementary Data File 2**). Conversely, there was significant normalised enrichment of the downregulated Kumar et al. signature genes in FNA and PBMC CD69- cells (**Supplementary Data File 2**). Performing GSEA using other gene sets relating to tissue residency further suggested FNA CD69+ cells as liver-resident T cells, however there was no significant enrichment in PBMC CD69+ cells. These data indicated that we were able to identify liver TRM cells transcriptionally, and differentiate them from liver TEM cells, using previously described residency signatures.

### Gene Expression Differences Between Liver TRM and TRM-Like Cells

**Figure 3:**
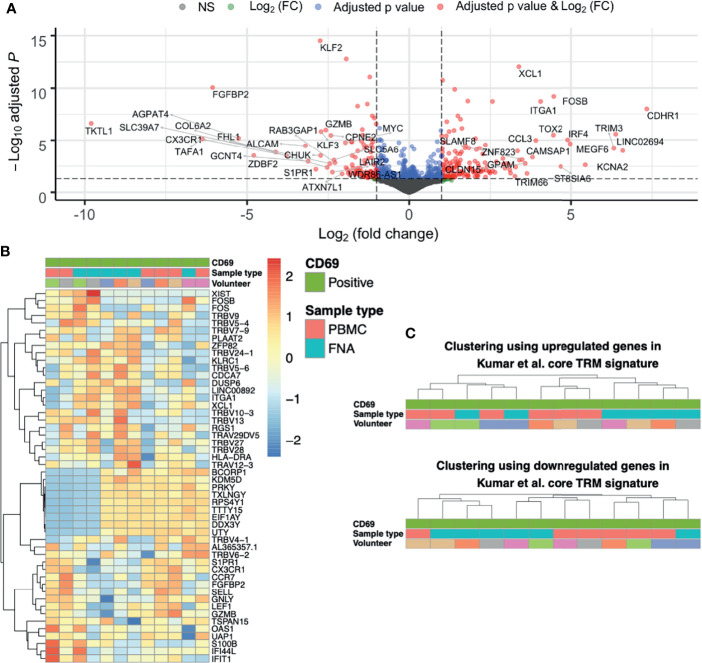
The differences between CD69+ CD8+ T cells isolated from liver and blood are unique and not like those when comparing TRM and TEM cells. **(A)** Volcano plot of up- and down-regulated genes between paired FNA CD69+ and PBMC CD69+ samples. The top 50 up- and down-regulated genes have been annotated. Positive log(FC) values indicate greater gene expression in FNA CD69+ samples, compared to PBMC CD69+ samples. Negative log(FC) values indicate greater gene expression in PBMC CD69+ samples. DGE of 11,933 genes was tested for significance using the Wald test. P values were adjusted using the Benjamini-Hochberg correction (false discovery rate). **(B)** Heatmap of the most variable genes in the FNA CD69+ vs PBMC CD69+ comparison. Fifty most variable genes between FNA CD69+ and PBMC CD69+ samples. Yellow/red corresponds to increased counts compared to other samples and blue corresponds to decreased counts. Genes selected according to those with the greatest variance across samples. Columns are clustered based on Spearman’s correlation of rlog normalised count values. Rows are clustered according to Pearson correlation of the rlog normalised gene counts. Gene count values are centred and scaled across rows. **(C)** Clustering of FNA and PBMC CD69+ samples using expression of TRM cell genes. Hierarchical clustering trees based on Spearman’s correlation of sample rlog normalised count values considering up- and down-regulated genes in CD8+ Kumar et al. core TRM cell transcriptional signature. FC, fold change; FNA, fine needle aspirate; PBMC, peripheral blood mononuclear cells; rlog, regularised logarithm; TRM, tissue-resident memory T cell.

Having shown that CD69+ identifies liver TRM cells both phenotypically and transcriptionally, we next compared gene expression difference between liver TRM and TRM-like cells. Overall, genes upregulated in TRM-like cells were involved in glucose metabolism (*TKTL1*), zinc transport (*SLC39A7*) and *de novo* phospholipid synthesis (*AGPAT4*), suggesting that TRM-like cells are metabolically active (**Figure 3A** and **Supplementary Data File 3**). In contrast, genes upregulated in TRM cells included those of inter-cellular communication (*XCL1*) and interferon-induced proteins (*TRIM3*). These data support the notion that TRM cells are metabolically quiescent and ready for rapid effector function upon activation (14, 41–43). Interestingly, in this comparison between TRM and TRM−like cells there was no differential expression of a set of genes that has previously been shown to differ between tissue−derived TEM and blood-derived TEM (23). Clustering based on the 50 most variable genes was unable to separate TRM and TRM-like cells and indicated a volunteer-specific effect, for nine Y chromosome−associated genes (**Figure 3B**). The TRM cell core signature proposed by Kumar et al. co-clustered TRM-like and TRM cells (**Figure 3C**), suggesting these two cells populations are more similar, in terms of these core residency genes, than liver TEM and TRM cells, which were clearly separated into two independent clusters (**Figure 2D**). Given this, we explored gene expression differences between TRM-like and TRM cells.

**Figure 4:**
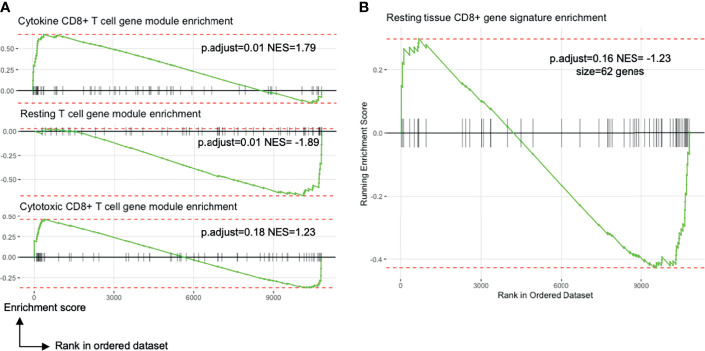
Blood TRM-like cells may not be only activated memory T cells. All plots show the comparison of liver TRM and blood TRM-like cells (see **Figure 2A**). **(A)** Enrichment plots of gene expression modules related to T cell transcriptional states identified by Szabo and colleagues. The CD8+ cytokine module includes genes encoding chemokines and cytokines (CCL3, CCL4, CCL20) and inhibitory molecules (LAG3, CD226, HAVCR2). The resting T cell module involves genes important for CD4+ and CD8+ T cell survival in blood and in tissues. The CD8+ cytotoxic module includes genes associated with cytotoxicity (GNLY, GZMK) and transcription factors associated with effector/memory differentiation (ZEB2, EOMES, ZNF683). NES is the enrichment score normalised to the mean enrichment of random samples of the same size. **(B)** Enrichment plot of a tissue CD8+ T cell signature identified by Szabo and colleagues (23). The complement of genes is derived by differential expression analyses between resting CCL5++ CD8+ memory T cells from several tissues compared to blood. FDR, false discovery rate; FNA, fine needle aspirate; GSEA, gene set enrichment analysis; GTPase, guanosine triphosphate hydrolase; MHC, major histocompatibility complex; ORA, over-representation analysis; p.adjust, Benjamini-Hochberg adjusted p value; PBMC, peripheral blood mononuclear cells.

Exploratory pathway analyses suggested differences between TRM and TRM−like cells. There was differential enrichment of genes involved in T cell differentiation, cell chemotaxis and interferon-gamma signalling (**Supplementary Figure 6A**). PBMC CD69+ T cells were enriched for gene sets related to GTPase activation, MHC protein complex binding, and ribosome structural component genes (**Supplementary Figure 6B**). Liver CD69+ T cells were enriched for genes involved in CD8+ T cell cytokine signalling (**Figure 4A**). There were no observed transcriptional differences in genes involved in cytotoxicity or a tissue-resident gene signature between liver CD69+ or PBMC CD69+ cells (**Figure 4B**). Other DGE analyses (**Figure 2A**) verified the uniqueness of TRM and TRM-like cells (**Supplementary Figures 6C–E**).

### Single-Cell RNA-Sequencing Elucidates Liver TRM Cell Subpopulations

**Figure 5:**
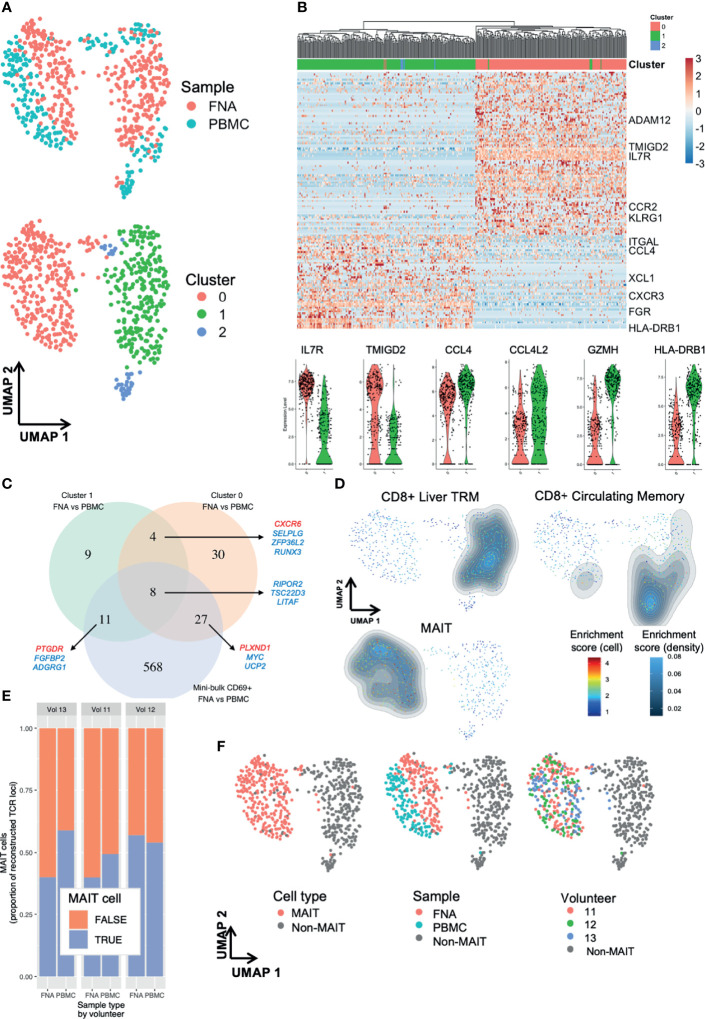
Single-cell RNA-sequencing reveals subpopulations of liver TRM and blood TRM-like cells. Single cell differences between TRM and TRM-like cells depend on TRM cell cluster. **(A)** UMAP plots of 629 single cells. Plots according to sample type and Seurat cluster. Seurat FindClusters was run at 0.4 resolution. Each point represents one cell, either a PBMC or FNA live single CD20- CD3+ CD45+ CD8+ CD4- CD45RA- CD69+ lymphocyte FACS sorted and sequenced by the SmartSeq2 protocol. Six hundred and twenty-nine cells are presented in these plots. **(B)** Gene heatmap of the C1 TRM vs C0 TRM cell comparison. Cell gene count is centred and scaled across all row values. A sample of genes are shown, a full list of the 142 genes differentially expressed between C1 and C0 TRM cells is available in the source data file. All genes shown here were present in at least 50% of cells in each cluster, had |ln(FC)|>0.5 and an adjusted p value (Bonferroni correction)<0.05. The row hierarchical clustering dendrogram is based on Euclidean distances of cell gene count values considering all genes. Violin plots of gene expression of six selected genes are presented below. The violin colour corresponds to FNA cell cluster of origin, as seen in **(A, C)** Venn diagram of genes differentiating TRM and TRM-like cells. Venn diagram demonstrating the degree of convergence of cluster-level DGE and mini-bulk-level DGE. All included genes from single cell contrasts had a |ln(FC)|>0.25 and an adjusted p value (Bonferroni correction)<0.05 and the genes from mini-bulk contrasts had |log2(FC)|>1 and an FDR < 0.05. DGE, differential gene expression. The mini-bulk contrast refers to that which is presented in **Figures 3**, **4**. A sample of the genes that are shared among the datasets is illustrated; blue indicates the gene is down-regulated in FNA CD69+ cells and red indicates the gene is up-regulated in FNA CD69+ cells, compared to blood CD69+ cells. **(D)** UMAP plots with signature enrichment scores. The enrichment of three signatures was assessed for each of the 629 cells and visualised in the UMAP by Single-Cell Signature Explorer (44). The per-cell signature enrichment was plotted, and density distribution of scores was overlaid. The signature scores represent a qualitative measure for visualisation. The signatures were obtained from Zhao et al. (22). **(E)** Proportion of recombinants that were derived from MAIT cells. MAIT cells were defined based on TCR α locus: any cell that expressed TRAV1-2 paired with TRAJ33, TRAJ12 or TRAJ20 was defined as a MAIT cell, regardless of the TCR β locus recombinant, if present. Any recombinant derived from a MAIT cell was labelled as such and excluded from non-MAIT cell analyses. **(F)** UMAP of 629 single cells with plots according to cluster, MAIT cell TCR, sample type and volunteer. In all plots, grey points (“non-MAIT”) represent cells that were either not classified as MAIT cells or cells for which TCR reconstruction could not be performed. Seurat FindClusters was run at 0.4 resolution, using a shared nearest neighbour clustering method. C, cluster; DGE, differential gene expression; FACS, fluorescence-assisted cell sorting; FDR, false discovery rate; FNA, fine needle aspirate; PBMC, peripheral blood mononuclear cells; MAIT, mucosal-associated invariant T cell; TRM, tissue-resident memory T cell; UMAP, uniform manifold approximation and projection.

Using single-cell RNA-sequencing (scRNA-seq) we were able to dissect the heterogeneity of liver TRM cell transcriptomes (**Figure 2A**) and identifed three main clusters (**Figure 5A** and **Supplementary Figures 7A, B**). There were 142 genes differentially expressed between TRM cells in cluster (C)1 and C0 (**Figure 5B** and **Supplementary Data File 4**). Notable differential expression included *HLA-D* locus, chemokine receptor and ligand, and mucosal-associated invariant T (MAIT) cell receptor genes (**Figure 5B**).

Comparing DGE between TRM and TRM-like cells in C1, C0 and the mini-bulk analysis, we saw some agreement between the lists (**Figure 5C**). This may indicate the signature obtained from mini-bulk RNA-seq was an amalgam of several transcriptomic profiles. C0 showed a clear demarcation between TRM and TRM-like cells (**Figure 5A**). Differentially expressed genes included increased expression of *KLF2*, *RUNX3* and *S1PR1* in TRM-like cells compared to increased expression of *CXCR6* and *CD69* in TRM cells in C0, despite all cells exhibiting surface expression of CD69 by flow cytometry (**Supplementary Data File 5**). The distribution of hepatic and blood cells in C1 appeared more heterogeneous and there were less differentially expressed genes (**Figure 5C** and **Supplementary Data File 6**). Using gene signatures derived from liver-resident CD8+ T cells and MAIT cells described by Zhao et al. (22), qualitative signature enrichment scores for each cell were represented on the UMAP (44). The liver TRM cell signature composed of 63 genes (such as *Gzmk*, *Cd160*, *Cxcr6* and *Eomes*) showed high enrichment scores in C1 FNA and PBMC-derived cells (**Figure 5D**). Both FNA and PBMC cells in C0 showed enrichment for the MAIT cell signature (**Figure 5D**). Interestingly, the circulating CD8+ memory T cell signature developed by Zhao et al. was highly enriched in C2 and, less so, in C1. Plausibly, C2 may have been composed of erroneously sorted CD69- cells or recently activated circulating CD69+ cells, particularly given the limited number of cells in this cluster. These signatures indicated C1 was comprised of cells resembling a conventional liver−resident T cell population and C0 was likely of MAIT or MAIT−like lineage cells.

TCR and CDR of the sequenced single cells were reconstructed using *in silico* techniques (45). There were several T cell clones that had frequencies of over 1% of reconstructed TCRs (**Supplementary Figure 8A**). Both FNA and PBMC samples exhibited substantial amounts of clonal and expanded clone T cells (**Supplementary Figures 8B, C**), as most expanded clones were present in both tissues. Similarly, almost all TCRα (TRAV & TRAJ) and TCRβ (TRBV & TRBJ) pairings in FNA samples were present in PBMC samples (**Supplementary Figure 8D**). Non-MAIT cell clones were present in both sample types, but no clones were shared between individuals (**Supplementary Figure 8E**). MAIT cells were present at considerable frequencies (**Figure 5E**). The proportion of MAIT cell recombinants did not vary greatly across volunteers or sample type (range 43-57%; **Figure 5E**). These analyses confirmed that most of C0 was composed of MAIT cells, with sparse contributions by MAIT cells to other clusters (**Figure 5F**). Importantly, MAIT cells were present in both sample types and in all three volunteers. On flow cytometry, the identified MAIT cells expressed higher quantities of CD69 and CD56, and lower CD11a and CD16 molecules, compared to non-MAIT cells (**Supplementary Figure 8F**). The TCR analyses suggested that there was shared clonality, TCRα and TCRβ chain usage, and MAIT cell frequencies between PBMC and FNA samples. Taken together, our transcriptomic analyses suggested a unique relationship between TRM and peripheral TRM-like cells.

### Predicting Vaccine-Induced Protection From Malaria Using Circulating TRM-Like Cells

**Figure 6:**
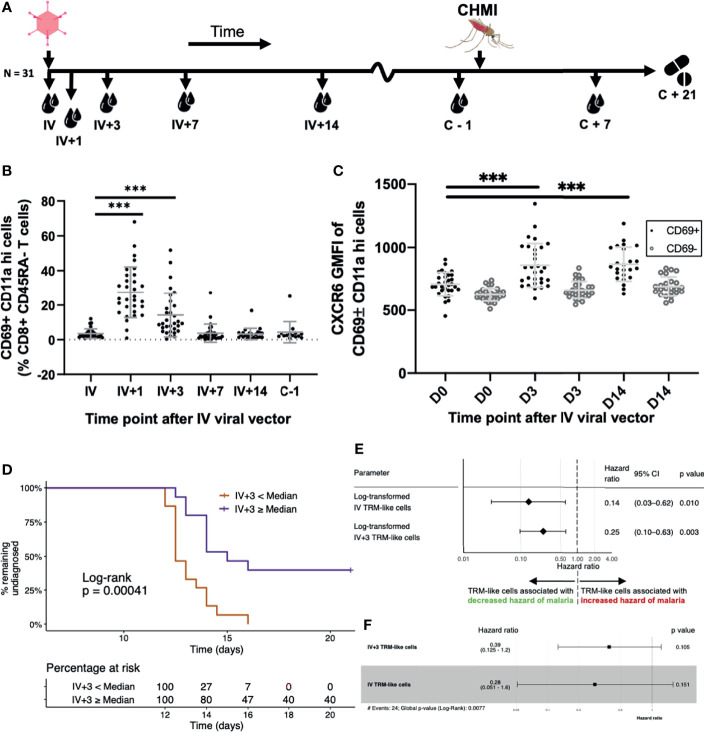
Blood TRM-like cells can be used to estimate the hazard of malaria diagnosis after CHMI. **(A)** Sampling workflow. Thirty-one volunteers across all vaccination groups were sampled at several time points following IV viral vector (IV+ timepoints) and around controlled human malaria infection. One volunteer from this cohort was not challenged. Five more control (non-vaccinated) volunteers were challenged and followed up at pre- and post-challenge time points, only. All volunteers were treated with standard anti-malarial therapy at day 21 post-CHMI or, if earlier, when they met our diagnostic criteria for malaria (see methods). **(B)** TRM-like cells after IV administration of viral vector. Frequency of circulating CD69+CD11ahi cells (as a proportion of CD8+CD45RA- cells) by time point. Comparisons were assessed using ratio paired t tests. Lines represent significant differences between the bound groups; bars show mean and standard deviation. **(C)** The GMFI of TRM-like cell CXCR6 at three different time points following IV viral vector. Comparisons were assessed using ratio paired t tests. Lines represent significant differences between the bound groups; bars show mean and standard deviation. Matched GMFI values of CD69- cells are also presented for reference. *** represents a p value < 0.0001. **(D)** Kaplan Meier curve using TRM-like cells 3 days after IV viral vector to stratify volunteers. Volunteers were stratified according to whether their TRM-like cell fraction (as a proportion of CD8+ CD45RA- cells) at day 3 after IV viral vector was above or below the median of all values. A log-rank test was performed to test for difference in survival (delay/lack of malaria diagnosis). The risk table shows the percentage of volunteers, in each stratum, at risk of malaria diagnosis at five representative time points. Right censoring occurred at 21 days as all undiagnosed volunteers received antimalarial therapy at this time. **(E)** Univariate Cox regression models using TRM−like cells [CD69+ CD11a hi frequency (% CD45RA- CD8+ CD3+ T cells)] measured at two time points. Each regression model estimated the effect that the variable had on an individual’s hazard of being diagnosed with malaria after CHMI. Hazard ratios less than one suggested that an increase in the TRM−like cell frequency decreased the instantaneous risk of malaria diagnosis over the study period. Hazard ratios greater than one suggested that a decrease in TRM−like cell frequency increased the instantaneous risk of malaria diagnosis over the study period. Log transformation was applied to the TRM−like cell frequency, and regression was performed on these values. The p value was calculated using a Wald test, with a null hypothesis that the parameter did not alter the hazard of malaria diagnosis after CHMI. **(F)** Multivariate Cox regression model using TRM like cells [CD69+ CD11a hi frequency (% CD45RA- CD8+ CD3+ T cells)] measured at two time points: IV and IV+3. Hazard ratios and 95%CI are presented. Log transformation was applied to the TRM−like cell frequency, and regression was performed on these values. The individual variable p values were calculated using a Wald test. The global p value was calculated using a Score (log−rank) test. Events refers to the number of volunteers that were diagnosed with malaria. AIC, Akaike information criterion; C, challenge; CHMI, controlled human malaria infection; D, day; GMFI, geometric mean fluorescence intensity; IV, intravenous viral vector administration; TRM, tissue-resident memory T cell.

Having demonstrated a relationship between liver TRM and peripheral TRM-like cells, we investigated the kinetics of TRM-like cells and the frequency of these cells as markers of liver-stage immunity as a potential correlate of vaccine-induced protection from malaria challenge. We performed flow cytometry on blood samples taken before a controlled human malaria infection (CHMI) study (**Figure 6A** and **Supplementary Figure 1**). The frequency of cells within the CD69+ CD11a hi subset of CD45RA- T cells peaked at day (D)1 after IV viral vector (IV+1) and remained elevated at IV+3, compared to day of IV viral vector administration (**Figure 6B** and **Supplementary Figures 9**, **10**, **11A–C**). Viral vector administration led to increased expression of liver TRM cell functional markers (eg. CXCR6), which remained elevated for at least two weeks post−vaccination compared to pre-vaccination levels (**Figure 6C** and **Supplementary Figure 11D**).

We next investigated whether the frequency of TRM-like cells in the blood prior to CHMI could be used to estimate a volunteer’s risk of malaria infection. Volunteers were stratified according to whether their IV+3 frequency of TRM-like cells was above or below the group median. Using this single parameter, we were able to separate volunteers who had a significantly greater rate of sterile protection (**Figure 6D**). IV+3 correlated with TRAP−specific T cells at several time points measured by flow cytometry or IFNγ ELISpot, with time to diagnosis and with mean parasites in the first three replication cycles (**Supplementary Figures 12A–E**). Only one other TRM-like cell parameter measured after IV vaccination, IV+1, had similar significance in estimating the risk of malaria diagnosis (**Supplementary Figure 12F**), but it did not correlate with IV+3 TRM-like cell frequency nor TRAP-specific T cells. Indeed, dichotomising volunteers according to TRAP-specific CD8+ T cells measured one day before CHMI did not separate volunteers with and without sterile protection (**Supplementary Figure 12F**).

Univariate and multivariate Cox models of TRM−like cells, and several other T cell populations, were generated to examine the effect of each predictor on the instantaneous risk of malaria diagnosis after CHMI. The two time points that were significantly associated with reduced hazard ratios (HR) were at IV vaccination (each log_10_ unit increase in TRM−like cells was associated with an 86% reduction in hazard of malaria diagnosis) and at IV+3 (each log_10_ unit increase in TRM−like cells was associated with a 75% reduction in hazard of malaria diagnosis; **Figure 6E**). Surprisingly, vaccine antigen-specific responses measured by ELISpot and ICS were not individually associated with a reduced hazard of malaria diagnosis, nor were other T cell subsets (**Supplementary Figure 13A**). The multivariate Cox model including TRM−like cells at IV and IV+3 was superior on Δ_𝑖_ analysis (46) compared to univariate Cox models composed of each variable alone (**Figure 6F**; concordance 0.723, Wald test p value = 0.0077). Both IV and IV+3 were robustly associated with a decreased hazard of malaria diagnosis (61% and 72% reduction per log unit increase, respectively), although individually in the multivariate analysis these variables were not significant (**Supplementary Figures 13B, C**). ELISpot and IFNγ+ (% CD8+, as measured by ICS) responses at several time points were included in forward stepwise model selection. The most parsimonious model Δ_𝑖_ analysis was composed of six parameters (**Supplementary Figure 13D**) but was composed of covariates that were correlated to varying degrees, resulting in multicollinearity. In addition, the parameters were taken from three different assays which, if these correlates were to be further examined in other, larger trials, presents practical difficulties. Lastly, for each model with less than five predictors, the most parsimonious model was composed entirely of TRM−like cell predictors. These results suggest that the benefits of including antigen-specific predictors into multivariate analysis did not outweigh the drawbacks of their inclusion. In this challenge study, we observed that increased frequency of TRM-like cells early post-vaccination correlated with higher levels of sterile protection from liver-stage malaria by infectious mosquito bite challenge. Overall these data would indicate that TRM-like cells are an indicator of bona-fide liver TRM cells, and therefore may be surrogate markers for liver-stage immunity.

## Discussion

Given the important role CD8+ T cells and TRM cells have been shown to play in protection from rodent malaria (9–11), an assay to measure TRM-associated cells in human peripheral blood could be a means of measuring liver TRM cells and, potentially, viral vector liver-stage malaria vaccine performance. In our study we identified a population of TRM-like cells in the circulation that correlated quantitatively and qualitatively to TRM cells isolated from the liver by FNA. Mini−bulk RNA-seq showed that although both cell populations displayed unique transcriptional properties, the differences were not a function of organ-specific differences and were substantively distinct to a core TRM cell signature seen by contrasting CD69+ TRM to CD69- TEM cells (14). scRNA-seq profiled these CD69+ TRM cells further, identified heterogeneous subpopulations and shared clonality between tissues. This work points to the potential for using CD69+ TRM-like cells in the periphery to estimate liver CD69+ TRM cells, and vaccine-induced protection from malaria. While our work is consistent with earlier studies indicating comparable frequencies of lymphocytes and that total CD3+ and CD8+ T cells positively correlate between PBMC and FNA samples (12, 30, 39), this study is the first to demonstrate bona fide TRM and TRM−like cells are related both quantitatively and qualitatively.

Previous reports indicate that major transcriptional differences between blood and tissue−residing T cells include apoptosis and cell-matrix interaction genes (23). Our data recapitulate some of these differences, but also shows that TRM and TRM-like cell differences are unique from other T cell comparisons across liver and blood. Pathway analyses indicated TRM-like cells were enriched for genes suggesting they were metabolically active but transcriptionally regulated, and prepared for leucocyte migration, when compared to liver TRM cells. These exploratory analyses, while not followed up with functional assays, could be the basis of further work examining blood CD69+ cells. Indeed, Walsh et al. have shown in mice that CD69 mediates recruitment and uptake of circulating CD8+ T cells in non−lymphoid tissues (47). Therefore, we hypothesise that TRM−like cells in the periphery circulate with a heightened predilection for tissue recruitment, and ability to follow chemotactic gradients, into the liver.

*In silico* reconstruction of single-cell TCRs confirmed the identity of MAIT cells and showed substantial clonal relationships between PBMC and FNA samples. The finding that clones coexisted and TCR chain sharing occurred between liver and blood is important, as it suggests that TRM-like cells were reflective of the liver TRM cell repertoire. MAIT cells are known to be highly abundant in the liver (48). This has been demonstrated using scRNA−seq gene expression (22) and TCR sequencing (49). MAIT cell oligoclonality among tissues and the circulation, and inter-individual homology have both been shown previously (49, 50). These TCR data were therefore in agreement with established literature regarding MAIT cells. However, this is the first report to comprehensively show the contribution of MAIT cells to the liver CD69+ memory CD8+ T cell compartment, indicating the underappreciated heterogeneity in previous reports defining traditional TRM cells as all hepatic CD69+ T cells (12, 17, 30, 51).

This work has a number of limitations. CD69 is well established as a very early activation marker (52) and it is possible that ‘TRM-like’ cells seen in the periphery, early after IV viral vector administration could merely be activated memory T cells. CD69 expression is known to decline six hours after activation (52), making it unlikely that the expression on memory T cells at IV+3 and later time points is entirely accounted for by an early inflammatory response. Despite significant evidence from animal models (9, 10, 53), the question as to whether TRMs are specifically induced by IV vaccination in humans remains an ongoing avenue of inquiry. We have seen that IV boosting induces substantially higher numbers of TRM-like cells, compared to IM boosting (data not shown). It is likely that only a proportion of the TRM-like cells identified in human peripheral blood i) were specific to the vaccine antigen and ii) established and maintained themselves in the liver long term. While bystander T cells lack specificity for the heterologous pathogen, they can however influence the immune response to infection, highlighting the importance of non-antigen-specific T cells (54). The pathway analyses presented were designed to be exploratory and could not provide conclusive evidence of functional and biological differences between TRM and TRM-like cells. These analyses could be the basis of future work examining TRM and TRM-like cells.

In this study we used FNAs to compare liver CD8+ TRM cells to circulating TRM-like counterparts, and highlight TRM-like cells as a potential surrogate marker of vaccine-induced protection against malaria. We found that while these peripheral ‘TRM-like’ cells differed to TRM cells in terms of significant pathways, such as leukocyte adhesion and T cell differentiation, they are phenotypically similar and indistinguishable in terms of T cell residency transcriptional signatures. Increases in the frequency of these TRM-like cells in the blood early after IV vaccination was associated with a reduced risk of developing malaria. This work provides proof-of-concept of multiple novel methods to investigate liver-stage malaria vaccines, immunological evaluation of a malaria vaccine strategy by CHMI, and insights into correlates of protection after vaccination. A simple, accessible and reproducible correlate of protection would be particularly helpful in trials of liver-stage malaria vaccines as they progress to phase III, large-scale testing in African infants. We provide a blueprint for understanding and monitoring liver TRM cells induced by a prime-target malaria vaccine approach.

## Data Availability Statement

The datasets presented in this article are not readily available in order to protect the privacy of the limited clinical trial participants and given the potentially re-identifiable nature of the data. Requests to access the datasets should be directed to author AVSH, adrian.hill@ndm.ox.ac.uk.

## Ethics Statement

The studies involving human participants were reviewed and approved by UK National Research Ethics Service, Committee South Central–Oxford REC A (18/SC/0384). The patients/participants provided their written informed consent to participate in this study.

## Author Contributions

Conception or design of the work: AN, AS, KE, AH. Acquisition, analysis and/or interpretation of data for the work: AN, MD, AF, DJ, DB, RAM, RM, FL, JS, AH, KE, AS, MH, DV, NP. Drafting the work or revising it critically for important intellectual content: AN, DV, NP, RM, AS, KE, AH. Provide approval for publication of the content: AN, AS, KE, AH. Agree to be accountable for all aspects of the work in ensuring that questions related to the accuracy or integrity of any part of the work are appropriately investigated and resolved: AN, MD, AF, DJ, DB, RAM, RM, FL, JS, AH, KE, AS, MH, DV, NP. All authors contributed to the article and approved the submitted version.

## Funding

This project has received funding from the European Union’s Horizon 2020 research and innovation programme under grant agreement No 733273. This research was funded by the National Institute for Health Research (NIHR) Oxford Biomedical Research Centre (BRC). This work was supported by the Medical Research Council [grant number MR/R015236/1]. AN was supported by The Rhodes Trust. This paper reflects the author’s view(s) and the European Commission are not responsible for any use that may be made of the information it contains. The views expressed are those of the author(s) and not necessarily those of the NHS, the NIHR or the Department of Health.

## Conflict of Interest

The authors declare that the research was conducted in the absence of any commercial or financial relationships that could be construed as a potential conflict of interest.

## Publisher’s Note

All claims expressed in this article are solely those of the authors and do not necessarily represent those of their affiliated organizations, or those of the publisher, the editors and the reviewers. Any product that may be evaluated in this article, or claim that may be made by its manufacturer, is not guaranteed or endorsed by the publisher.

## Supplementary Material

Supplementary Figure 1 Trial overview and timeline. Detailed schematic of trial. The final study cohort included 35 volunteers, across five groups, with group sizes for the first four (intervention) groups as indicated. Not shown in this figure is the infectivity control group (N=5) that did not receive any vaccinations and underwent CHMI with the vaccinated groups. All intervention group volunteers received either two or three vaccines of viral vectors encoding ME-TRAP. Doses were as indicated. Fine needle aspirates occurred between 16 and 24 days after IV vaccination/boost. Controlled Human Malaria Infection (CHMI, challenge) of volunteers by infected mosquitoes was carried out in a CL3 suite. Volunteers underwent CHMI on two separate days (4/2/2019 and 5/2/2019) and participants were equally and randomly divided across these days. Infectious mosquitos were provided by the Walter Reed Army Institute of Research. Volunteers were exposed to the bites of five infectious mosquitoes per participant for 5-10 minutes. Fed mosquitoes were individually dissected and assessed for sporozoite load, to ensure all fed mosquitoes were infected with P. falciparum. Volunteers were followed up twice per day from five days after challenge. Volunteers were treated with 20mg artemether/120mg lumefantrine (Riamet) upon malaria diagnosis or 21 days after challenge. A diagnosis of malaria infection was made given the following criteria: 1) The presence of symptoms suggestive of malaria in addition to a qPCR result indicating ≥ 1,000 parasites/mL OR 2) Lack of symptoms with a qPCR result indicating ≥ 10,000 parasites/mL. None of the volunteers in the infectivity control group achieved sterile protection.

Supplementary Figure 2 Gating strategy for paired liver FNA and PBMC samples. Boxes on flow cytometry plots define the representative sequential gating strategy identifying TRM in human liver FNA and TRM-like cells in human PBMC. Singlets, CD45+, live CD20-, lymphocytes, CD3+, CD8+/CD4+, CD56-, CD69+ CD45RA ± CD11a hi ± CD103. FNA, fine needle aspirate; FSC-A/H, forward scatter; LIVE/DEAD Aqua, fixable dead cell stain; PBMC, peripheral blood mononuclear cells; SSC-A, side scatter.

Supplementary Figure 3 Representative flow cytometry plots from FNA and PBMC of the same donors. Boxes on flow cytometry plots define the representative gating strategy identifying several T cells subsets in human liver FNA and PBMC. Percentages are expressed relative to total singlet, CD45+, live, CD20-, CD3+, CD8+/CD4+, CD56- T cells. Previous gating prior to this included: singlets, CD45+, live CD20-, lymphocytes, CD3+, CD8+/CD4+, CD56-. FNA, fine needle aspirate; PBMC, peripheral blood mononuclear cells.

Supplementary Figure 4 Expression of TRM markers on FNA TRM and PBMC TRM-like cells. TRM/TRM-like cell expression of the selected markers was compared to paired (within volunteer and within sample) TEM (CD69-) expression of the selected marker. Both the positive fraction of cells and the GMFI of the selected markers is presented. Two-way ANOVA results are presented below each plot. Pairing by tissue and CD69 status was performed. Equal variability of differences was assumed when performing repeated measures two-way ANOVA (based on GLM). ANOVA, Analysis of variance. FNA, fine needle aspirate. GLM, general linear model. PBMC, peripheral blood mononuclear cells.

Supplementary Figure 5 FNA and PBMC t-distributed stochastic neighbour embedding (tSNE). The tSNE algorithm was run using single cell expression values of 13 markers on 1,068,990 cells pre-gated on CD45+ single live lymphocyte size. These lymphocytes were derived from all 13 volunteers. Each FNA sample contributed on average 32,760 cells (range 6480-50,000), while each PBMC sample contributed 50,000 cells. **(A)** Contour tSNE plot of FNA and PBMC CD8+ and CD8+ TRM cells. Five distinct clusters of TRM can be seen. (B) Contour tSNE plot of TRM/TRM-like cells compared to TEM (non-TRM) from both tissues. The left plot shows only those cells derived from the liver (FNA), while the right shows those from the blood (PBMC). **(C)** Contour tSNE plot of TRM/TRM-like cells from both tissues. The gates of the three tSNE subsets are shown with the corresponding percentage of total cells. **(D)** Phenotype and metaparameter characterisation of the three tSNE subsets. The three subsets can be differentiated by PD-1, CXCR6 and CD103 expression. The tSNE plot shows the positions of the three subsets and the left histograms show the GMFI of the listed markers for the three subsets. The three subsets have been selected due to their over-representation in CD69+ memory T cells compared to CD69- memory T cells. The right histograms show the relative distribution of cells into discrete experimental and technical categories. tSNE subsets are relatively equally spread across each of the metaparameters. Metaparameters were not included when running the tSNE algorithm. FNA, fine needle aspirate. GMFI, geometric mean fluorescence intensity. PBMC, peripheral blood mononuclear cells. tSNE, t-distributed stochastic neighbour embedding.

Supplementary Figure 6 Pathway analyses. **(A)** Over-representation analysis plots for gene ontology categories of the FNA CD69+ vs PBMC CD69+ comparison. A: Over-representation analysis gene-concept network plots for enriched Gene Ontology biological process and molecular function categories. A hyper geometric test was used to test for significance of gene category over-representation. Gene categories presented have an FDR < 0.05. Size refers to the number of genes involved in category. **(B)** Enrichment plots for gene ontology categories of the FNA CD69+ vs PBMC CD69+ comparison. Gene set enrichment analyses visualised by category running enrichment score among pre-ranked list of all 11,933 differentially expressed genes. A category’s enrichment score is the maximum deviation from zero encountered in the running score. If the normalised enrichment score is positive, this suggests enrichment of the category in FNA CD69+ samples. A negative normalised enrichment score suggests enrichment of the category in PBMC CD69+. The ranked list metric is the binary logarithm of the fold change of gene expression between FNA CD69+ and PBMC CD69+ cells. **(C)** Venn diagram of the FNA CD69- vs PBMC CD69- comparison. Venn diagram demonstrating the overlap of genes that are in both FNA vs PBMC comparisons (where CD69+ cells are compared, left blue circle, and CD69- cells, right orange circle). All genes were differentially expressed with an FDR < 0.01. Ten genes co−occurred in the differential expression lists generated by comparing FNA and PBMC CD69+, and FNA and PBMC CD69- (not significant, hypergeometric test p≃1). **(D)** GSEA plots of the FNA CD69- vs PBMC CD69- comparison. Gene set enrichment analyses of gene ontologies visualised by category running enrichment score among pre-ranked list of all 12,062 differentially expressed genes. A category’s enrichment score is approximated by the maximum deviation, from zero, encountered in the running score. If the normalised enrichment score is positive, this suggests enrichment of the category in FNA CD69- samples. A negative normalised enrichment score suggests enrichment of the category in PBMC CD69-. All categories presented had an FDR < 0.05. Considering gene expression differences between PBMC CD69+ and CD69- samples, no terms were enriched with FDR<0.05 on GSEA when using Hallmark MSigDB gene sets or gene ontology sets (data not shown). **(E)** Clustering of the samples in the PBMC CD69+ vs CD69- comparison. Hierarchical clustering tree based on Spearman’s correlation of sample rlog normalised count values considering all genes in CD8+ Kumar et al. core TRM transcriptional signature. FDR, false discovery rate. FNA, fine needle aspirate. GSEA, gene set enrichment analysis. PBMC, peripheral blood mononuclear cells. rlog, regularised logarithm. TRM, tissue-resident memory T cell.

Supplementary Figure 7 PCA plots based on the 500 most variable genes in the single cell RNA sequencing experiment. Each point represents one cell, either a PBMC or FNA live single CD20- CD3+ CD45+ CD8+ CD4- CD45RA- CD69+ lymphocyte FACS sorted and sequenced in plates by the SmartSeq2 protocol. Six hundred and twenty-nine cells are presented in these PC plots. Cell cycle phase was assigned using the cyclone function from the scran package version 1.14.6. **(A)** Plots of the first two PC, according to various technical and biological parameters. PC1 is associated with the number of features per cell (SCT normalisation in Seurat was performed). PC2 does not appear to be substantially separated by the presented parameters. **(B)** Plots of PC2 and PC3, according to various technical and biological parameters. PC3 appears to separate PBMC and FNA cells. FACS, fluorescence-assisted cell sorting. FC, flow cell. G1, gap 1 cell cycle phase. G2, gap 2 cell cycle phase. mtDNA, mitochondrial DNA PC, principal component. PCA, principal component analysis. S, synthesis cell cycle phase. V, volunteer.

Supplementary Figure 8 T cell clonality and clone size. **(A)** Out of the 690 cells analysed, productive TCR α and β loci were reconstructed in 634 (91.9%) and 639 (92.6%) cells, respectively. There was productive reconstruction of both α and β loci from 603 (87.4%) cells. Most cells had one productive α or β locus identified (Table 4.4). Relatively few cells had two productive α or β loci (Table 4.4), **(B)** TCR alpha (left) and beta (right) loci clone frequency. Bars represent individual clones and bar height represents clone frequency. A clone was defined as any number of cells that had the exact same (alpha or beta) TCR loci and CDR3 nucleotide sequence. Clones with more than two cells are displayed here. Non-productive recombinants and recombinants derived from MAIT cells were excluded from these analyses. Clone sizes are reported as a frequency, in relation to the total number of reconstructed TCR recombinants. **(C)** Clonality by volunteer and sample type. Alpha and beta TCR loci recombinants categorised according to whether they were expanded (shared by more than two cells), clonal (present in two cells) or unique (present in one cell). **(D)** TCR alpha and beta chain usage of expanded clonal T cells by sample type. Chain pairing of TRAV & TRAJ and TRBV & TRBJ are displayed as chord diagrams where ribbons connecting arcs indicate frequency of chain pairing. Only clones of greater than two cells and productive recombinants were included in these analyses. MAIT cells were excluded from these analyses. Ticks on the inside of the arcs are present for every 5 cells that shared the recombinant. Recombinant totals: alpha chain usage FNA n=75, PBMC n=30; beta chain usage FNA n=51, PBMC n=17. The degree of TCR chain usage sharing between samples is representative of all volunteers. **(E)** Clonal relationships between MAIT and non-MAIT cells. The number of recombinants shared between MAIT (left) and non-MAIT (right) cells. Nodes (vertices) represent individual cells and links (edges) represent a unique recombinant shared between the connected cells. Cells could share up to a maximum of four recombinants (two TCR α and two TCR β). Cells are distributed using a force-directed layout algorithm whereby cells have a simulated ‘charge’, repelling them from other cells, and links have attractive forces for connected cells. For a given number of cells, more links resulted in tighter clusters. A clone was defined as any number of cells that had the exact same (α or β) TCR loci and CDR3 nucleotide sequence. Clones with more than two cells are displayed here. MAIT cells were defined based on TCR α locus: any cell that expressed TRAV1-2 paired with TRAJ33, TRAJ12 or TRAJ20 was defined as a MAIT cell, regardless of the TCR β locus recombinant. **(F)** Flow cytometry index sort information obtained at the time of single-cell isolation for CD69, CD56, CD11a and CD16 molecules. Values represent fluorochrome geometric mean fluorescence intensity that has been compensated, but not transformed. FNA, fine needle aspirate. MAIT, mucosal associated invariant T cell. PBMC, peripheral blood mononuclear cell. recomb(s), TCR recombinant. TCR, T cell receptor. TCRA, T cell receptor alpha locus. TCRB, T cell receptor beta locus. Vol, volunteer.

Supplementary Figure 9 TRM-like cells gating strategy. Boxes on flow cytometry plots define the representative sequential gating strategy identifying TRM-like cells in human PBMC. Singlets, lymphocytes, live, (CD4 & CD20)-, CD3+, CD8+, CD45RA- and CD69+ CD11a hi using 16-color flow cytometry. FSC-A/H, forward scatter; LIVE/DEAD Aqua, fixable dead cell stain; PBMC, peripheral blood mononuclear cells; SSC-A, side scatter.

Supplementary Figure 10 Representative flow cytometry plots from representative two volunteers samples at different time points. Boxes on flow cytometry plots define the representative gating strategy identifying TRM like cells in PBMC. Percentages are expressed relative to total singlet, live, (CD4 & CD20)-, CD3+, CD8+, CD45RA-T cells. Previous gating prior to this included: singlets, lymphocytes, live, (CD4 & CD20)-, CD3+, CD8+, CD45RA- and CD69+ CD11a hi using 16-colour flow cytometry. PBMC, peripheral blood mononuclear cells.

Supplementary Figure 11 TRM like cells after IV vaccination. **(A)** CD69+ CD11a hi cells as proportions of CD8+ CD3+ T cells at six time points after IV vaccination. **(B)** CD69+ CD11a hi cells as proportions of CD45RA- CD8+ CD3+ T cells by time after IV vaccination. For each time point, there was a window in which volunteers could attend for blood sampling. **(C)** CD69+ CD11a hi CD45RA- CD8+ CD3+ T cell concentration calculated using volunteers’ clinical haematology value for total lymphocytes. Mean values are presented, and error bars represent the standard deviation. Significance testing of differences between time points was performed using ratio paired t tests and the resultant p values of all tested comparisons are shown (unless specifically mentioned). An α of less than 0.05 was considered statistically significant. These analyses were performed by assuming that 30% of lymphocytes were CD8+ CD3+, as has been previously demonstrated in Caucasian UK and US populations (!ref,!ref). **(D)** Ki67+ frequencies of CD69+ and CD69- CD11a hi CD45RA- CD8+ CD3+ T cells (left), and Ki67 geometric mean fluorescence intensities of CD69+ and CD69- CD11a hi CD45RA- CD8+ CD3+ T cells (right). CD69- (effector memory T cell) frequency and expression of the markers are presented as an internal comparator. All comparisons between CD69+ and CD69- cells were significant (p<0.001). Significance testing of differences between individual groups was performed using ratio paired t-tests, and the resultant p values of all tested comparisons are shown. Statistics were performed on non-transformed data.

Supplementary Figure 12 IV+3 TRM−like cells vs indicators of vaccine-induced immunity and protection.**(A–C)** X-axes and values are shared between all plots. Empty grey triangles represent volunteers that achieved sterile protection and were removed from the “without protection” analyses. **(A)** TRM−like cell frequencies; CD69+ CD11a hi CD45RA- (% CD8+ CD3+ T cells) cell frequencies, at IV+3, compared to IFNγ ELISpot AgSp T cell response at C-1 (left), C+35 (middle) and C+90 (right). **(B)** TRM−like cell frequencies at IV+3, compared to IFNγ+ cell (% CD8+ CD3+ T cells) response at C-1 (left) and C+7 (right). **(C)** TRM−like cell frequencies at IV+3, compared to the maximum IFN−γ ELISpot AgSp T cell response (left) and the maximum IFNγ+ cell (% CD8+ CD3+ T cells) response as measured by ICS (right). IFN−γ ELISpot was performed at between 10 and 13 time points, and ICS was performed at three time points. Spearman’s rank correlation coefficient, the correlation coefficient 95% confidence interval and an approximate, unadjusted p value are reported. **(D)** TRM−like cell frequencies (CD69+ CD11a hi CD45RA- (% CD8+ CD3+ T cells)) measured at IV+3 compared across volunteers who experienced any (sterile or partial) vaccine-induced protection and those with no protection. Partial protection was defined as volunteers with a diagnosis of malaria 14 days or later, after infectious mosquito bite [!ref] **(E)** Associations of TRM−like cell frequencies at IV+3, and time to malaria diagnosis (left) and the mean number of parasites in the first three replication cycles (right). Empty grey triangles represent volunteers that were right-censored from the study and deemed to have achieved sterile protection from malaria. These volunteers did not reach 1000 blood-stage parasites, therefore were not included in this analysis. Spearman’s rank correlation coefficient, the correlation coefficient 95% confidence interval and an approximate, unadjusted p value are reported. Statistics were performed on non-transformed data. For log-transformed PCR data, a pseudocount value of one was added to each qPCR value to allow log transformation of zero values (y=log(1+qPCR)). **(F)** Kaplan−Meier curve comparisons using TRM−like cells one day after IV vaccination. Volunteers were stratified according to whether their CD69+ CD11a hi CD45RA- frequency (% CD8+ CD3+ T cells) above or below the median of all values. A log−rank test was performed to test for difference in survival (delay/lack of malaria diagnosis), and the p value is reported in the plot. The risk table shows the percentage of volunteers, in each stratum, at risk of malaria diagnosis at five representative time points. Right censoring occurred at 21 days as all undiagnosed volunteers received antimalarial therapy. Time, on the x-axes, refers to the time after CHMI. Kaplan Meier curve comparisons using AgSp responses at C-1 to stratify volunteers: IFNγ ELISpot ME-TRAP SFC per million PBMC (left) and IFNγ+ frequency (% CD8+) as measured by intracellular cytokine staining (right).

Supplementary Figure 13
**(A)** Univariate Cox regression models using antigen-specific responses: IFNγ ELISpot ME-TRAP SFC per million PBMC (left) and IFNγ+ frequency (% CD8+) as measured by intracellular cytokine staining (right). **(B)** Univariate Cox regression models using non-antigen-specific T cell subsets: CD69- CD11a hi CD45RA- CD8+ CD3+ T cells (left) and CD45RA- CD8+ CD3+ T cells (right). **(C)** Schoenfeld residuals plotted to test the proportional hazards assumption (top). Systematic departures from a horizontal line when plotting Schoenfeld residuals would indicate non-proportional hazards. The assumption of proportional hazards was supported for both covariates and overall, for the model (p=0.304). The blue dashed lines are fit by local linear regression (lowess), to aid in interpretation; the shading represents ± 2-standard-error envelopes around the fit. The red dashed line is at y=0. C: Dfbetas plotted to assess influential observations. Plotting dfbetas estimated the changes in regression coefficients upon deleting each observation in turn (bottom). Comparing the magnitudes of the largest dfbeta values to the regression coefficients suggests that none of the observations were largely influential. The model satisfied the proportional hazards assumption (p=0.304 overall) and there were no vastly influential observations. IV and IV+3 TRM−like cell parameters were previously shown to be moderately positively correlated (p=3.433x10-4,, ). The variance inflation factor, which measures the inflation in variance due to parameter correlation, of both variables was 1.38. **(D)** Akaike information criterion values of models composed of all permutations of ten log-transformed TRM−like cell, IFNγ+ (%CD8+ as measured by ICS) and IFNγ ELISpot parameters.

Supplementary Data Sheet 1 Differentially expressed gene list, adjusted p values and shrunken log2 fold change values for FNA CD69+ vs FNA CD69- comparison.

Supplementary Data Sheet 2 Results of gene set enrichment analysis using custom gene sets obtained from Kumar et al., Zhao et al. and Szabo et al.

Supplementary Data Sheet 3 Differentially expressed gene list, adjusted p values and shrunken log2 fold change values for FNA CD69+ vs PBMC CD69+ comparison.

Supplementary Data Sheet 4 Differentially expressed gene list, adjusted p values and average natural log fold change values for cluster 0 FNA CD69+ vs cluster 1 FNA CD69+ single cell comparison.

Supplementary Data Sheet 5 Differentially expressed gene list, adjusted p values and average natural log fold change values for cluster 0 FNA CD69+ vs cluster 0 PBMC CD69+ single cell comparison.

Supplementary Data Sheet 6 Differentially expressed gene list, adjusted p values and average natural log fold change values for cluster 1 FNA CD69+ vs cluster 1 PBMC CD69+ single cell comparison.
