# Supplementary figures and images for: Deep Immune Phenotyping and Single-Cell Transcriptomics Allow Identification of Circulating TRM-Like Cells Which Correlate With Liver-Stage Immunity and Vaccine-Induced Protection From Malaria
Source: Front Immunol. 2022 Feb 7;13:795463. doi: 10.3389/fimmu.2022.795463 (PMC8859435; doi:10.3389/fimmu.2022.795463)

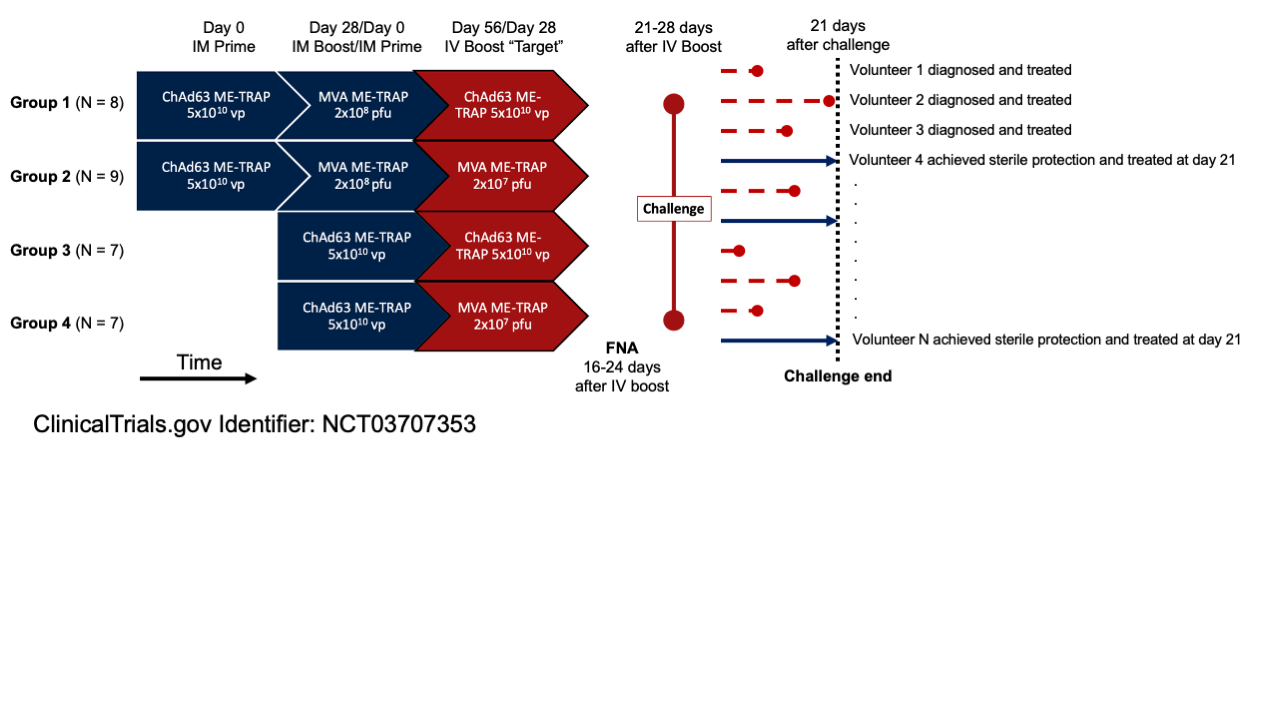

Supplement: Supplementary Figure 1 — Trial overview and timeline. Detailed schematic of trial. The final study cohort included 35 volunteers, across five groups, with group sizes for the first four (intervention) groups as indicated. Not shown in this figure is the infectivity control group (N=5) that did not receive any vaccinations and underwent CHMI with the vaccinated groups. All intervention group volunteers received either two or three vaccines of viral vectors encoding ME-TRAP. Doses were as indicated. Fine needle aspirates occurred between 16 and 24 days after IV vaccination/boost. Controlled Human Malaria Infection (CHMI, challenge) of volunteers by infected mosquitoes was carried out in a CL3 suite. Volunteers underwent CHMI on two separate days (4/2/2019 and 5/2/2019) and participants were equally and randomly divided across these days. Infectious mosquitos were provided by the Walter Reed Army Institute of Research. Volunteers were exposed to the bites of five infectious mosquitoes per participant for 5-10 minutes. Fed mosquitoes were individually dissected and assessed for sporozoite load, to ensure all fed mosquitoes were infected with P. falciparum. Volunteers were followed up twice per day from five days after challenge. Volunteers were treated with 20mg artemether/120mg lumefantrine (Riamet) upon malaria diagnosis or 21 days after challenge. A diagnosis of malaria infection was made given the following criteria: 1) The presence of symptoms suggestive of malaria in addition to a qPCR result indicating ≥ 1,000 parasites/mL OR 2) Lack of symptoms with a qPCR result indicating ≥ 10,000 parasites/mL. None of the volunteers in the infectivity control group achieved sterile protection. [file DataSheet_2.zip › Supplementary Figure 1.tiff]

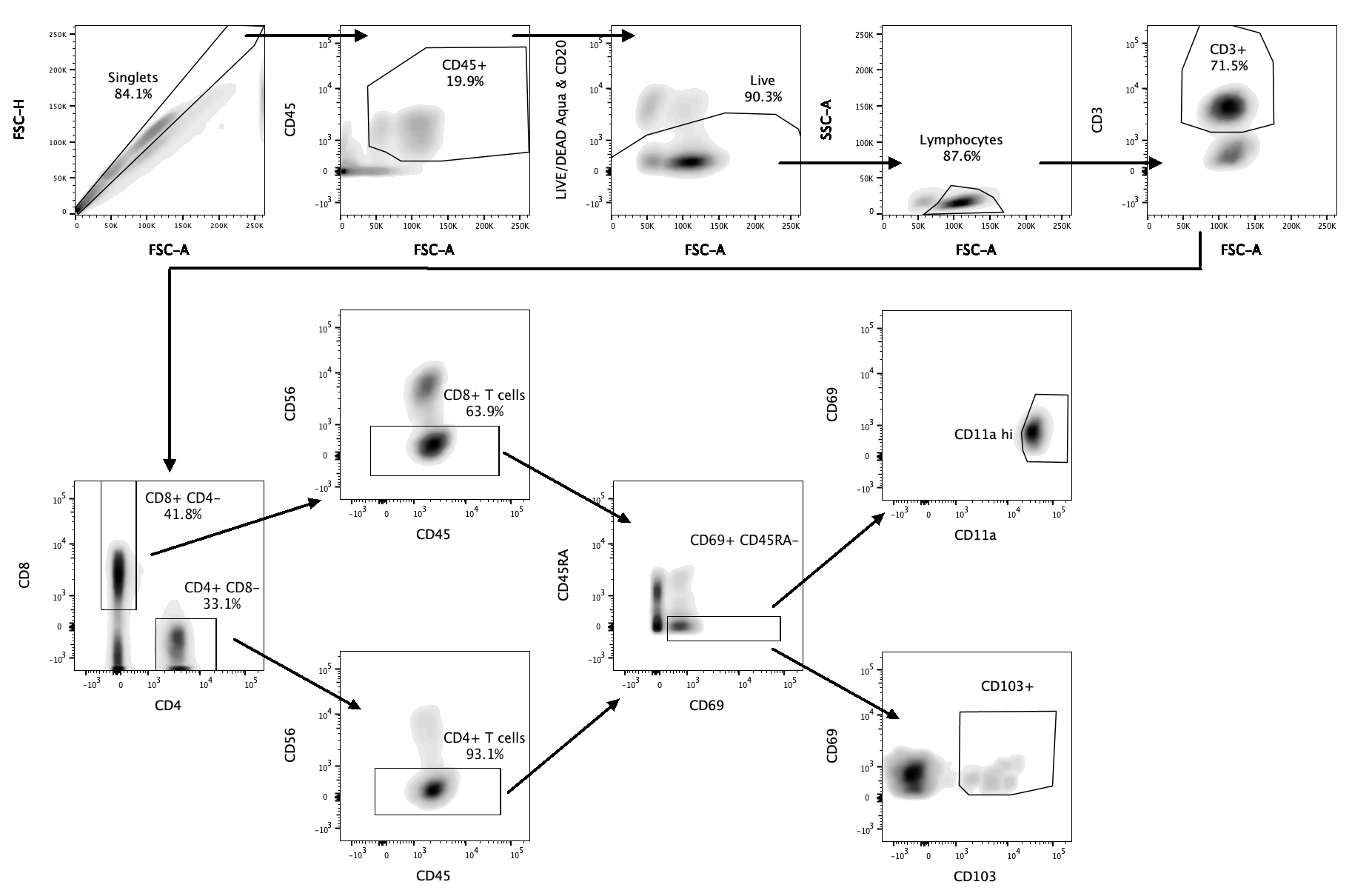

Supplement: Supplementary Figure 1 — Trial overview and timeline. Detailed schematic of trial. The final study cohort included 35 volunteers, across five groups, with group sizes for the first four (intervention) groups as indicated. Not shown in this figure is the infectivity control group (N=5) that did not receive any vaccinations and underwent CHMI with the vaccinated groups. All intervention group volunteers received either two or three vaccines of viral vectors encoding ME-TRAP. Doses were as indicated. Fine needle aspirates occurred between 16 and 24 days after IV vaccination/boost. Controlled Human Malaria Infection (CHMI, challenge) of volunteers by infected mosquitoes was carried out in a CL3 suite. Volunteers underwent CHMI on two separate days (4/2/2019 and 5/2/2019) and participants were equally and randomly divided across these days. Infectious mosquitos were provided by the Walter Reed Army Institute of Research. Volunteers were exposed to the bites of five infectious mosquitoes per participant for 5-10 minutes. Fed mosquitoes were individually dissected and assessed for sporozoite load, to ensure all fed mosquitoes were infected with P. falciparum. Volunteers were followed up twice per day from five days after challenge. Volunteers were treated with 20mg artemether/120mg lumefantrine (Riamet) upon malaria diagnosis or 21 days after challenge. A diagnosis of malaria infection was made given the following criteria: 1) The presence of symptoms suggestive of malaria in addition to a qPCR result indicating ≥ 1,000 parasites/mL OR 2) Lack of symptoms with a qPCR result indicating ≥ 10,000 parasites/mL. None of the volunteers in the infectivity control group achieved sterile protection. [file DataSheet_2.zip › Supplementary Figure 2.png]

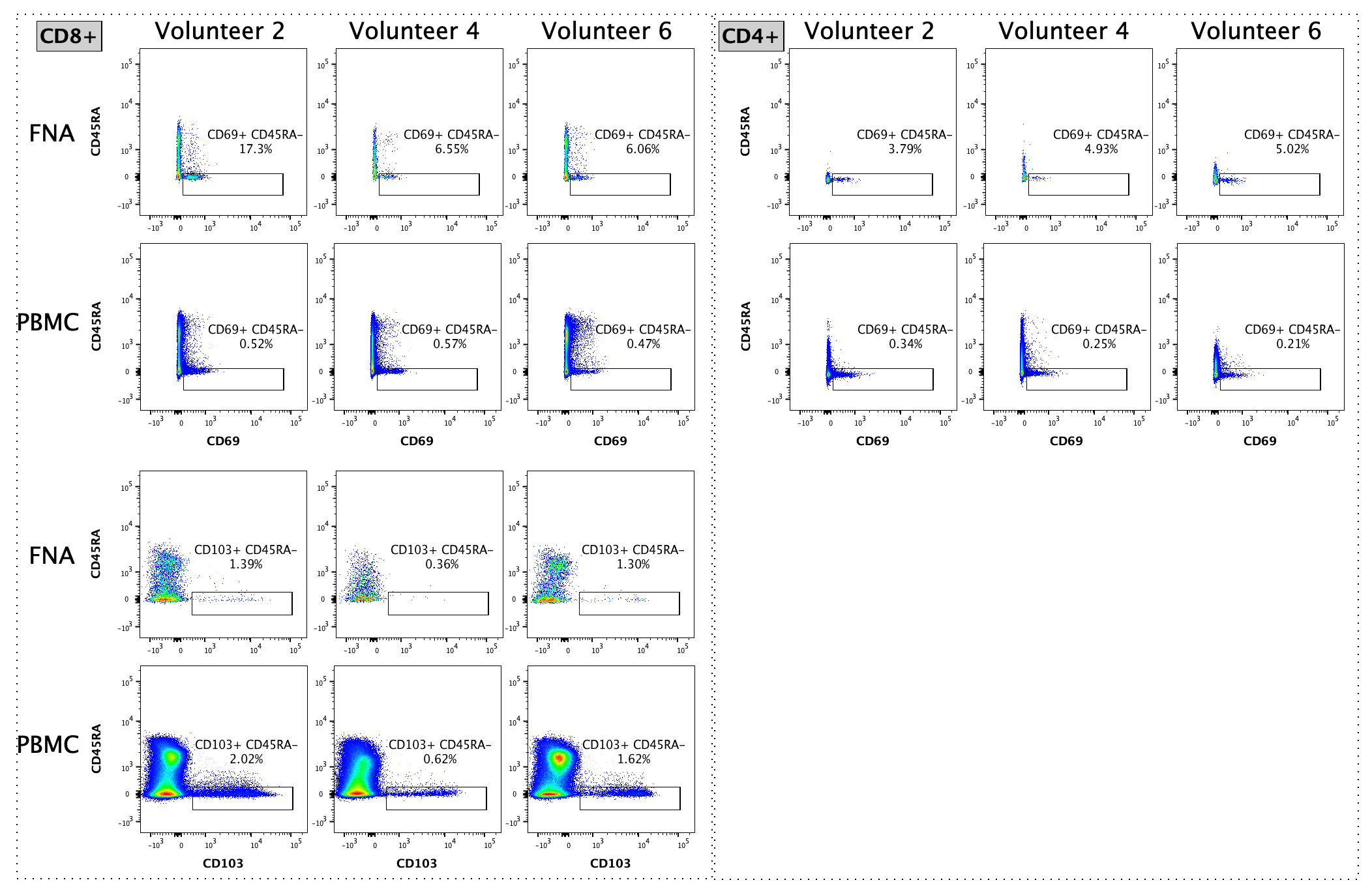

Supplement: Supplementary Figure 1 — Trial overview and timeline. Detailed schematic of trial. The final study cohort included 35 volunteers, across five groups, with group sizes for the first four (intervention) groups as indicated. Not shown in this figure is the infectivity control group (N=5) that did not receive any vaccinations and underwent CHMI with the vaccinated groups. All intervention group volunteers received either two or three vaccines of viral vectors encoding ME-TRAP. Doses were as indicated. Fine needle aspirates occurred between 16 and 24 days after IV vaccination/boost. Controlled Human Malaria Infection (CHMI, challenge) of volunteers by infected mosquitoes was carried out in a CL3 suite. Volunteers underwent CHMI on two separate days (4/2/2019 and 5/2/2019) and participants were equally and randomly divided across these days. Infectious mosquitos were provided by the Walter Reed Army Institute of Research. Volunteers were exposed to the bites of five infectious mosquitoes per participant for 5-10 minutes. Fed mosquitoes were individually dissected and assessed for sporozoite load, to ensure all fed mosquitoes were infected with P. falciparum. Volunteers were followed up twice per day from five days after challenge. Volunteers were treated with 20mg artemether/120mg lumefantrine (Riamet) upon malaria diagnosis or 21 days after challenge. A diagnosis of malaria infection was made given the following criteria: 1) The presence of symptoms suggestive of malaria in addition to a qPCR result indicating ≥ 1,000 parasites/mL OR 2) Lack of symptoms with a qPCR result indicating ≥ 10,000 parasites/mL. None of the volunteers in the infectivity control group achieved sterile protection. [file DataSheet_2.zip › Supplementary Figure 3.png]

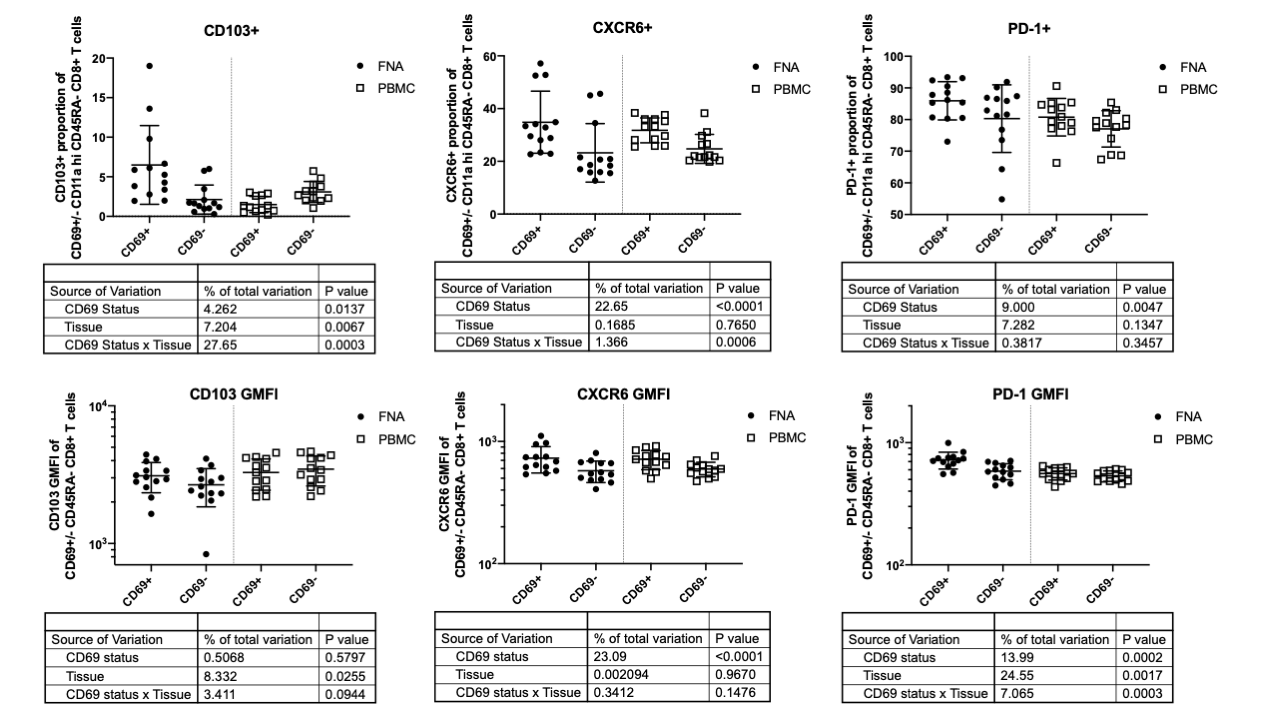

Supplement: Supplementary Figure 1 — Trial overview and timeline. Detailed schematic of trial. The final study cohort included 35 volunteers, across five groups, with group sizes for the first four (intervention) groups as indicated. Not shown in this figure is the infectivity control group (N=5) that did not receive any vaccinations and underwent CHMI with the vaccinated groups. All intervention group volunteers received either two or three vaccines of viral vectors encoding ME-TRAP. Doses were as indicated. Fine needle aspirates occurred between 16 and 24 days after IV vaccination/boost. Controlled Human Malaria Infection (CHMI, challenge) of volunteers by infected mosquitoes was carried out in a CL3 suite. Volunteers underwent CHMI on two separate days (4/2/2019 and 5/2/2019) and participants were equally and randomly divided across these days. Infectious mosquitos were provided by the Walter Reed Army Institute of Research. Volunteers were exposed to the bites of five infectious mosquitoes per participant for 5-10 minutes. Fed mosquitoes were individually dissected and assessed for sporozoite load, to ensure all fed mosquitoes were infected with P. falciparum. Volunteers were followed up twice per day from five days after challenge. Volunteers were treated with 20mg artemether/120mg lumefantrine (Riamet) upon malaria diagnosis or 21 days after challenge. A diagnosis of malaria infection was made given the following criteria: 1) The presence of symptoms suggestive of malaria in addition to a qPCR result indicating ≥ 1,000 parasites/mL OR 2) Lack of symptoms with a qPCR result indicating ≥ 10,000 parasites/mL. None of the volunteers in the infectivity control group achieved sterile protection. [file DataSheet_2.zip › Supplementary Figure 4.tiff]

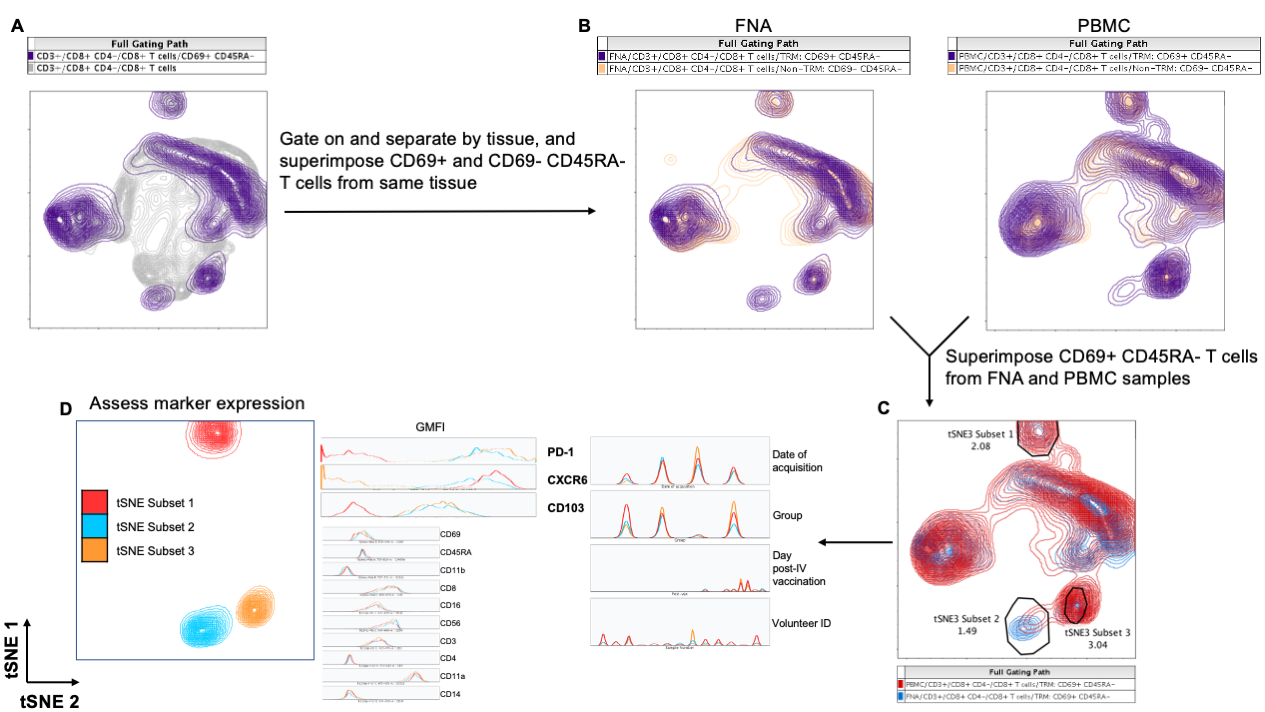

Supplement: Supplementary Figure 1 — Trial overview and timeline. Detailed schematic of trial. The final study cohort included 35 volunteers, across five groups, with group sizes for the first four (intervention) groups as indicated. Not shown in this figure is the infectivity control group (N=5) that did not receive any vaccinations and underwent CHMI with the vaccinated groups. All intervention group volunteers received either two or three vaccines of viral vectors encoding ME-TRAP. Doses were as indicated. Fine needle aspirates occurred between 16 and 24 days after IV vaccination/boost. Controlled Human Malaria Infection (CHMI, challenge) of volunteers by infected mosquitoes was carried out in a CL3 suite. Volunteers underwent CHMI on two separate days (4/2/2019 and 5/2/2019) and participants were equally and randomly divided across these days. Infectious mosquitos were provided by the Walter Reed Army Institute of Research. Volunteers were exposed to the bites of five infectious mosquitoes per participant for 5-10 minutes. Fed mosquitoes were individually dissected and assessed for sporozoite load, to ensure all fed mosquitoes were infected with P. falciparum. Volunteers were followed up twice per day from five days after challenge. Volunteers were treated with 20mg artemether/120mg lumefantrine (Riamet) upon malaria diagnosis or 21 days after challenge. A diagnosis of malaria infection was made given the following criteria: 1) The presence of symptoms suggestive of malaria in addition to a qPCR result indicating ≥ 1,000 parasites/mL OR 2) Lack of symptoms with a qPCR result indicating ≥ 10,000 parasites/mL. None of the volunteers in the infectivity control group achieved sterile protection. [file DataSheet_2.zip › Supplementary Figure 5.tiff]

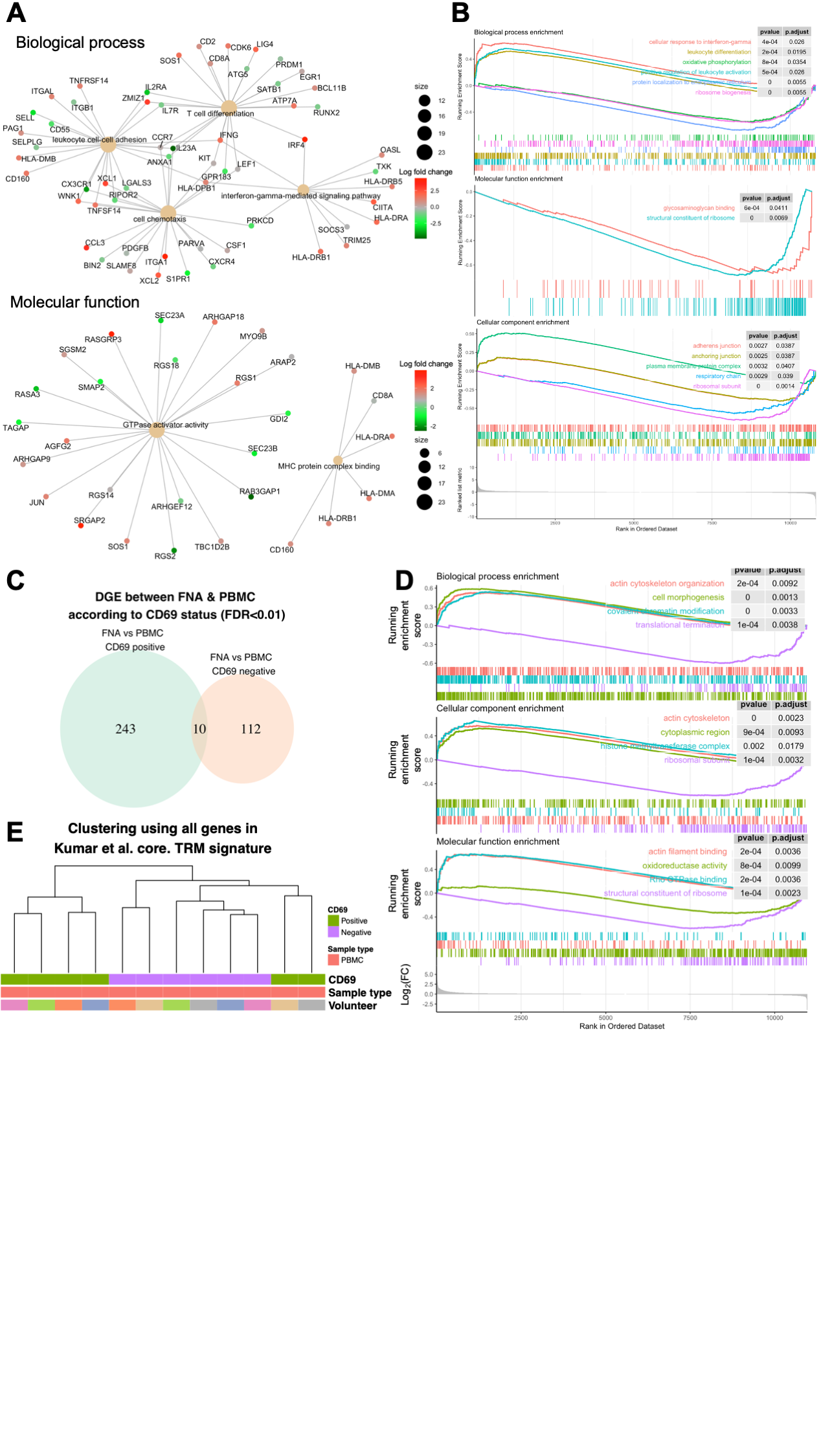

Supplement: Supplementary Figure 1 — Trial overview and timeline. Detailed schematic of trial. The final study cohort included 35 volunteers, across five groups, with group sizes for the first four (intervention) groups as indicated. Not shown in this figure is the infectivity control group (N=5) that did not receive any vaccinations and underwent CHMI with the vaccinated groups. All intervention group volunteers received either two or three vaccines of viral vectors encoding ME-TRAP. Doses were as indicated. Fine needle aspirates occurred between 16 and 24 days after IV vaccination/boost. Controlled Human Malaria Infection (CHMI, challenge) of volunteers by infected mosquitoes was carried out in a CL3 suite. Volunteers underwent CHMI on two separate days (4/2/2019 and 5/2/2019) and participants were equally and randomly divided across these days. Infectious mosquitos were provided by the Walter Reed Army Institute of Research. Volunteers were exposed to the bites of five infectious mosquitoes per participant for 5-10 minutes. Fed mosquitoes were individually dissected and assessed for sporozoite load, to ensure all fed mosquitoes were infected with P. falciparum. Volunteers were followed up twice per day from five days after challenge. Volunteers were treated with 20mg artemether/120mg lumefantrine (Riamet) upon malaria diagnosis or 21 days after challenge. A diagnosis of malaria infection was made given the following criteria: 1) The presence of symptoms suggestive of malaria in addition to a qPCR result indicating ≥ 1,000 parasites/mL OR 2) Lack of symptoms with a qPCR result indicating ≥ 10,000 parasites/mL. None of the volunteers in the infectivity control group achieved sterile protection. [file DataSheet_2.zip › Supplementary Figure 6.tiff]

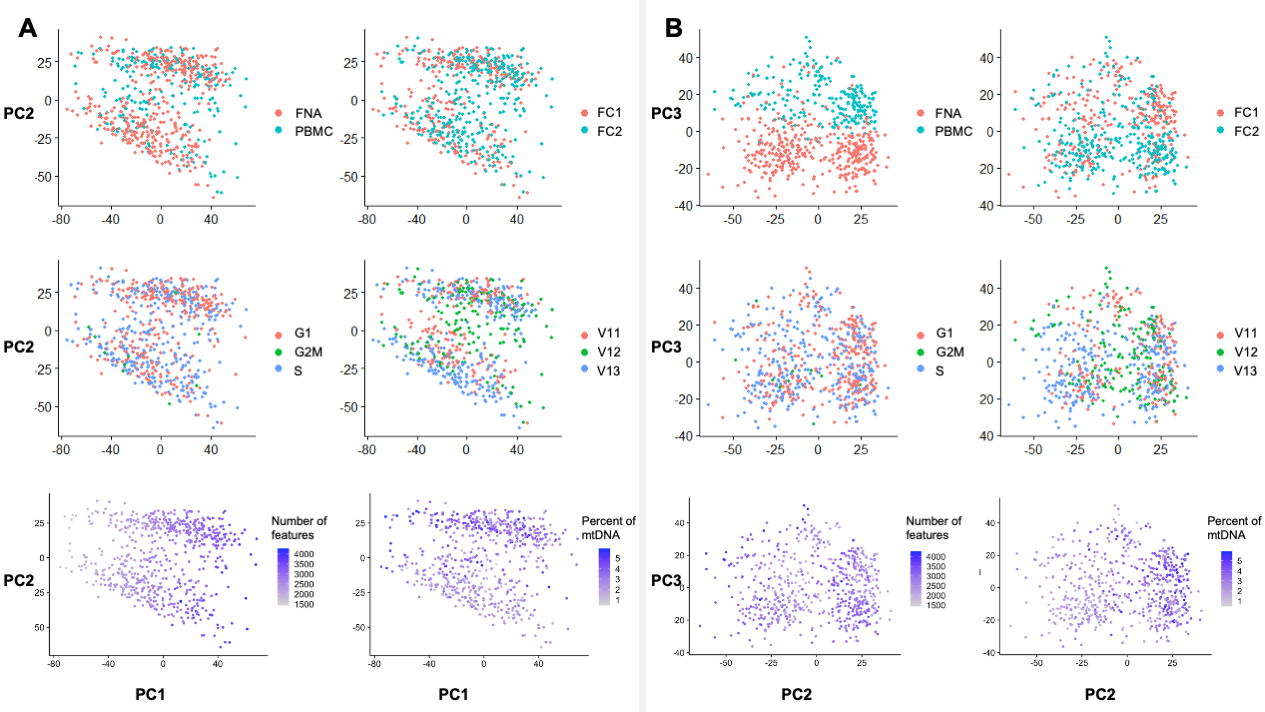

Supplement: Supplementary Figure 1 — Trial overview and timeline. Detailed schematic of trial. The final study cohort included 35 volunteers, across five groups, with group sizes for the first four (intervention) groups as indicated. Not shown in this figure is the infectivity control group (N=5) that did not receive any vaccinations and underwent CHMI with the vaccinated groups. All intervention group volunteers received either two or three vaccines of viral vectors encoding ME-TRAP. Doses were as indicated. Fine needle aspirates occurred between 16 and 24 days after IV vaccination/boost. Controlled Human Malaria Infection (CHMI, challenge) of volunteers by infected mosquitoes was carried out in a CL3 suite. Volunteers underwent CHMI on two separate days (4/2/2019 and 5/2/2019) and participants were equally and randomly divided across these days. Infectious mosquitos were provided by the Walter Reed Army Institute of Research. Volunteers were exposed to the bites of five infectious mosquitoes per participant for 5-10 minutes. Fed mosquitoes were individually dissected and assessed for sporozoite load, to ensure all fed mosquitoes were infected with P. falciparum. Volunteers were followed up twice per day from five days after challenge. Volunteers were treated with 20mg artemether/120mg lumefantrine (Riamet) upon malaria diagnosis or 21 days after challenge. A diagnosis of malaria infection was made given the following criteria: 1) The presence of symptoms suggestive of malaria in addition to a qPCR result indicating ≥ 1,000 parasites/mL OR 2) Lack of symptoms with a qPCR result indicating ≥ 10,000 parasites/mL. None of the volunteers in the infectivity control group achieved sterile protection. [file DataSheet_2.zip › Supplementary Figure 7.tiff]

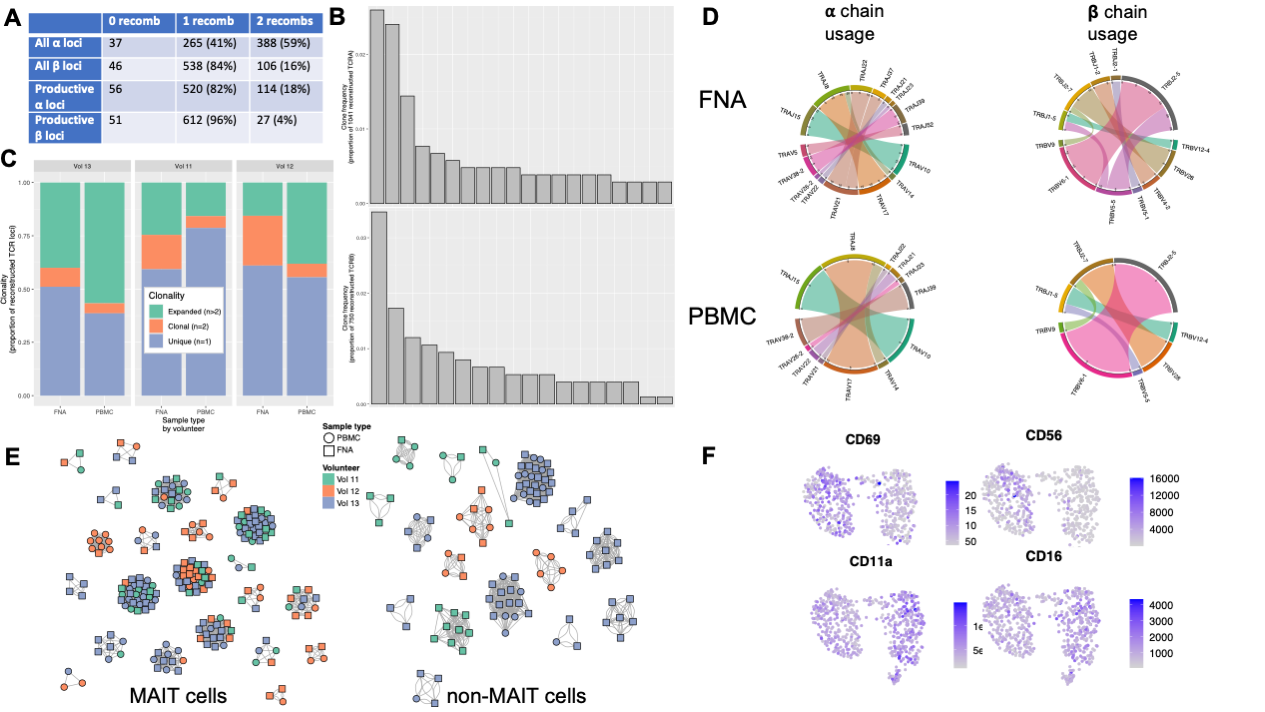

Supplement: Supplementary Figure 1 — Trial overview and timeline. Detailed schematic of trial. The final study cohort included 35 volunteers, across five groups, with group sizes for the first four (intervention) groups as indicated. Not shown in this figure is the infectivity control group (N=5) that did not receive any vaccinations and underwent CHMI with the vaccinated groups. All intervention group volunteers received either two or three vaccines of viral vectors encoding ME-TRAP. Doses were as indicated. Fine needle aspirates occurred between 16 and 24 days after IV vaccination/boost. Controlled Human Malaria Infection (CHMI, challenge) of volunteers by infected mosquitoes was carried out in a CL3 suite. Volunteers underwent CHMI on two separate days (4/2/2019 and 5/2/2019) and participants were equally and randomly divided across these days. Infectious mosquitos were provided by the Walter Reed Army Institute of Research. Volunteers were exposed to the bites of five infectious mosquitoes per participant for 5-10 minutes. Fed mosquitoes were individually dissected and assessed for sporozoite load, to ensure all fed mosquitoes were infected with P. falciparum. Volunteers were followed up twice per day from five days after challenge. Volunteers were treated with 20mg artemether/120mg lumefantrine (Riamet) upon malaria diagnosis or 21 days after challenge. A diagnosis of malaria infection was made given the following criteria: 1) The presence of symptoms suggestive of malaria in addition to a qPCR result indicating ≥ 1,000 parasites/mL OR 2) Lack of symptoms with a qPCR result indicating ≥ 10,000 parasites/mL. None of the volunteers in the infectivity control group achieved sterile protection. [file DataSheet_2.zip › Supplementary Figure 8.tiff]

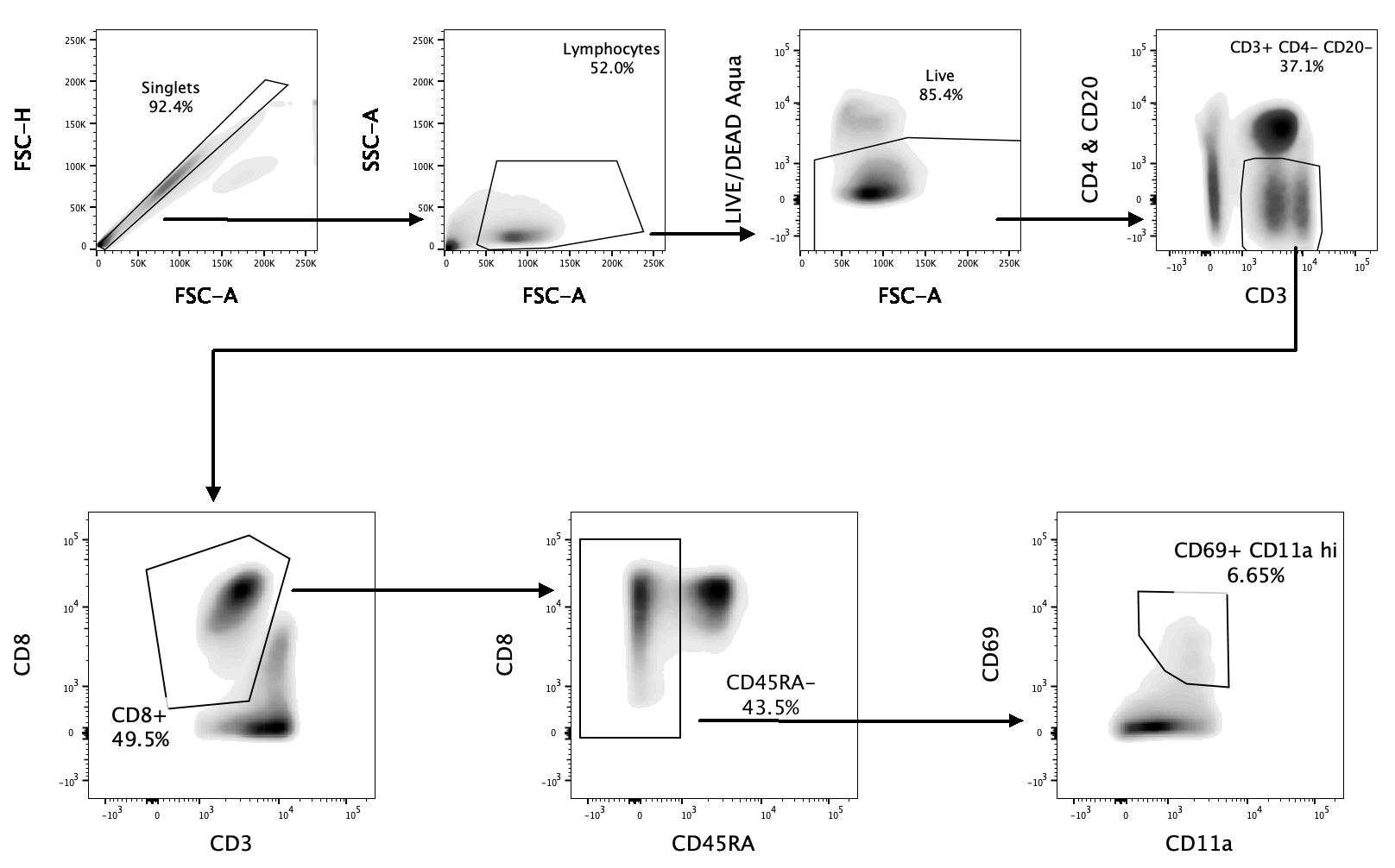

Supplement: Supplementary Figure 1 — Trial overview and timeline. Detailed schematic of trial. The final study cohort included 35 volunteers, across five groups, with group sizes for the first four (intervention) groups as indicated. Not shown in this figure is the infectivity control group (N=5) that did not receive any vaccinations and underwent CHMI with the vaccinated groups. All intervention group volunteers received either two or three vaccines of viral vectors encoding ME-TRAP. Doses were as indicated. Fine needle aspirates occurred between 16 and 24 days after IV vaccination/boost. Controlled Human Malaria Infection (CHMI, challenge) of volunteers by infected mosquitoes was carried out in a CL3 suite. Volunteers underwent CHMI on two separate days (4/2/2019 and 5/2/2019) and participants were equally and randomly divided across these days. Infectious mosquitos were provided by the Walter Reed Army Institute of Research. Volunteers were exposed to the bites of five infectious mosquitoes per participant for 5-10 minutes. Fed mosquitoes were individually dissected and assessed for sporozoite load, to ensure all fed mosquitoes were infected with P. falciparum. Volunteers were followed up twice per day from five days after challenge. Volunteers were treated with 20mg artemether/120mg lumefantrine (Riamet) upon malaria diagnosis or 21 days after challenge. A diagnosis of malaria infection was made given the following criteria: 1) The presence of symptoms suggestive of malaria in addition to a qPCR result indicating ≥ 1,000 parasites/mL OR 2) Lack of symptoms with a qPCR result indicating ≥ 10,000 parasites/mL. None of the volunteers in the infectivity control group achieved sterile protection. [file DataSheet_2.zip › Supplementary Figure 9.png]

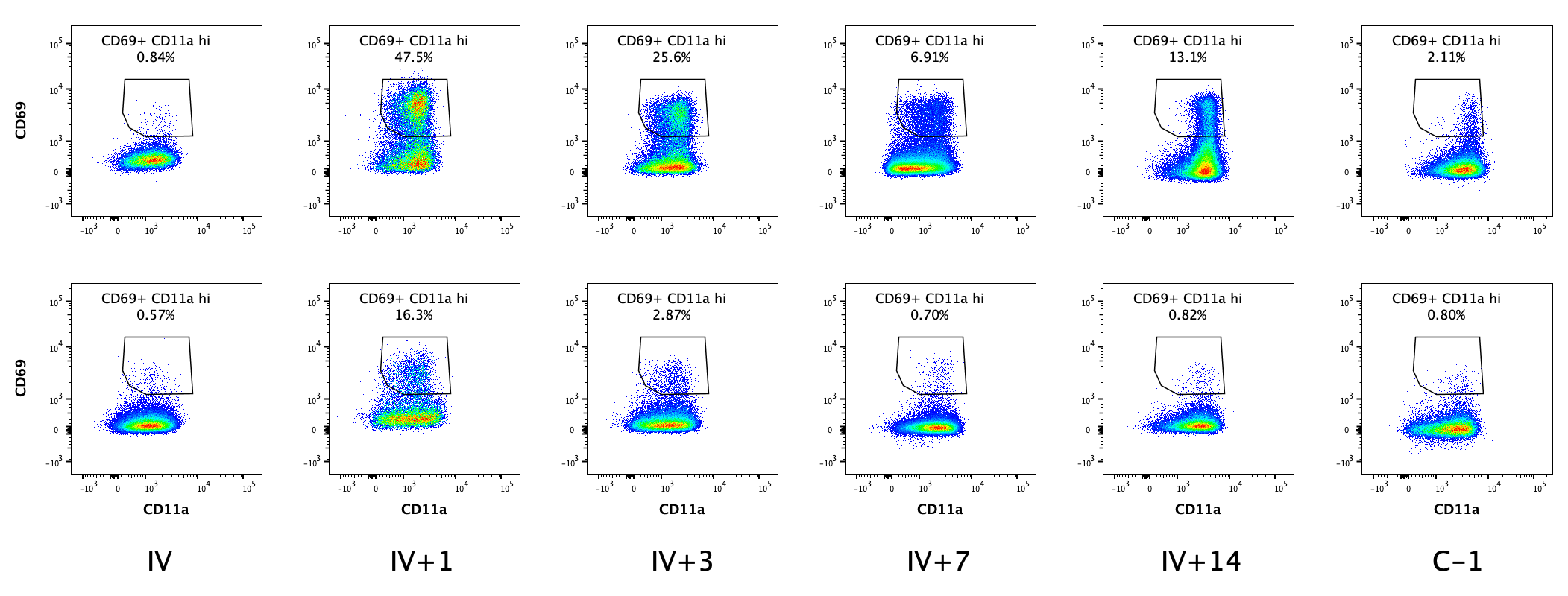

Supplement: Supplementary Figure 1 — Trial overview and timeline. Detailed schematic of trial. The final study cohort included 35 volunteers, across five groups, with group sizes for the first four (intervention) groups as indicated. Not shown in this figure is the infectivity control group (N=5) that did not receive any vaccinations and underwent CHMI with the vaccinated groups. All intervention group volunteers received either two or three vaccines of viral vectors encoding ME-TRAP. Doses were as indicated. Fine needle aspirates occurred between 16 and 24 days after IV vaccination/boost. Controlled Human Malaria Infection (CHMI, challenge) of volunteers by infected mosquitoes was carried out in a CL3 suite. Volunteers underwent CHMI on two separate days (4/2/2019 and 5/2/2019) and participants were equally and randomly divided across these days. Infectious mosquitos were provided by the Walter Reed Army Institute of Research. Volunteers were exposed to the bites of five infectious mosquitoes per participant for 5-10 minutes. Fed mosquitoes were individually dissected and assessed for sporozoite load, to ensure all fed mosquitoes were infected with P. falciparum. Volunteers were followed up twice per day from five days after challenge. Volunteers were treated with 20mg artemether/120mg lumefantrine (Riamet) upon malaria diagnosis or 21 days after challenge. A diagnosis of malaria infection was made given the following criteria: 1) The presence of symptoms suggestive of malaria in addition to a qPCR result indicating ≥ 1,000 parasites/mL OR 2) Lack of symptoms with a qPCR result indicating ≥ 10,000 parasites/mL. None of the volunteers in the infectivity control group achieved sterile protection. [file DataSheet_2.zip › Supplementary Figure 10.png]

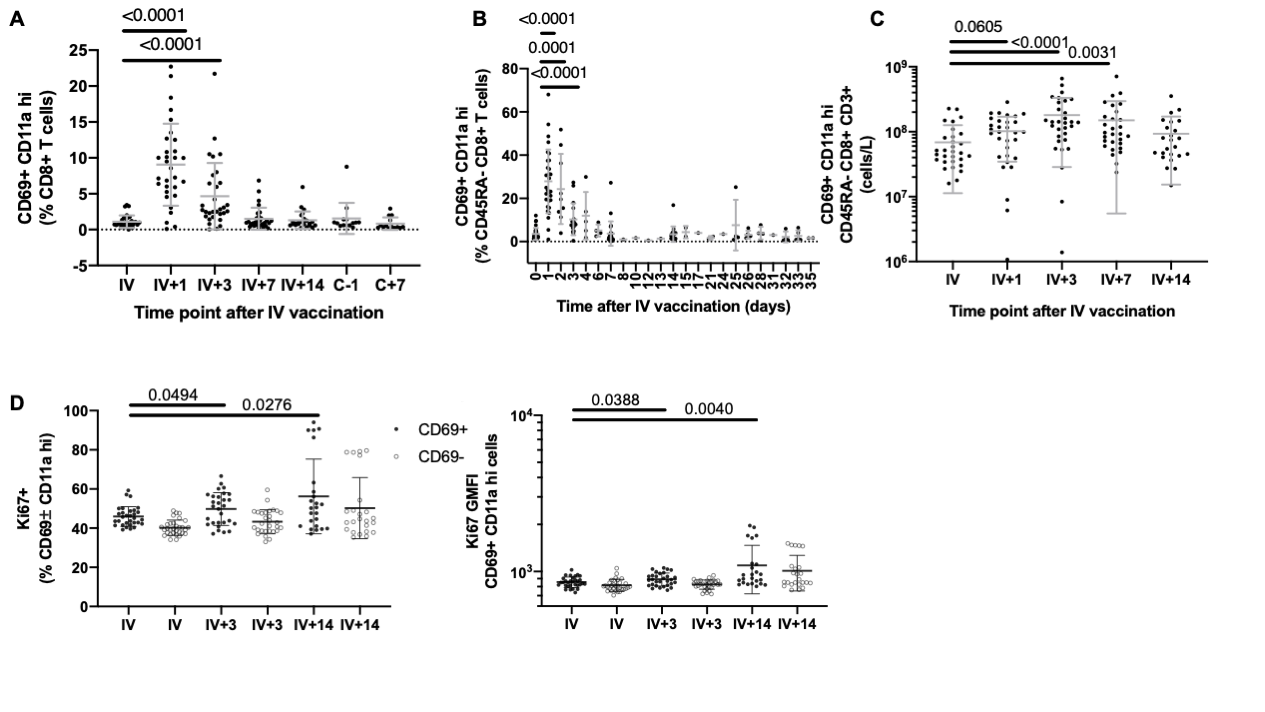

Supplement: Supplementary Figure 1 — Trial overview and timeline. Detailed schematic of trial. The final study cohort included 35 volunteers, across five groups, with group sizes for the first four (intervention) groups as indicated. Not shown in this figure is the infectivity control group (N=5) that did not receive any vaccinations and underwent CHMI with the vaccinated groups. All intervention group volunteers received either two or three vaccines of viral vectors encoding ME-TRAP. Doses were as indicated. Fine needle aspirates occurred between 16 and 24 days after IV vaccination/boost. Controlled Human Malaria Infection (CHMI, challenge) of volunteers by infected mosquitoes was carried out in a CL3 suite. Volunteers underwent CHMI on two separate days (4/2/2019 and 5/2/2019) and participants were equally and randomly divided across these days. Infectious mosquitos were provided by the Walter Reed Army Institute of Research. Volunteers were exposed to the bites of five infectious mosquitoes per participant for 5-10 minutes. Fed mosquitoes were individually dissected and assessed for sporozoite load, to ensure all fed mosquitoes were infected with P. falciparum. Volunteers were followed up twice per day from five days after challenge. Volunteers were treated with 20mg artemether/120mg lumefantrine (Riamet) upon malaria diagnosis or 21 days after challenge. A diagnosis of malaria infection was made given the following criteria: 1) The presence of symptoms suggestive of malaria in addition to a qPCR result indicating ≥ 1,000 parasites/mL OR 2) Lack of symptoms with a qPCR result indicating ≥ 10,000 parasites/mL. None of the volunteers in the infectivity control group achieved sterile protection. [file DataSheet_2.zip › Supplementary Figure 11.tiff]

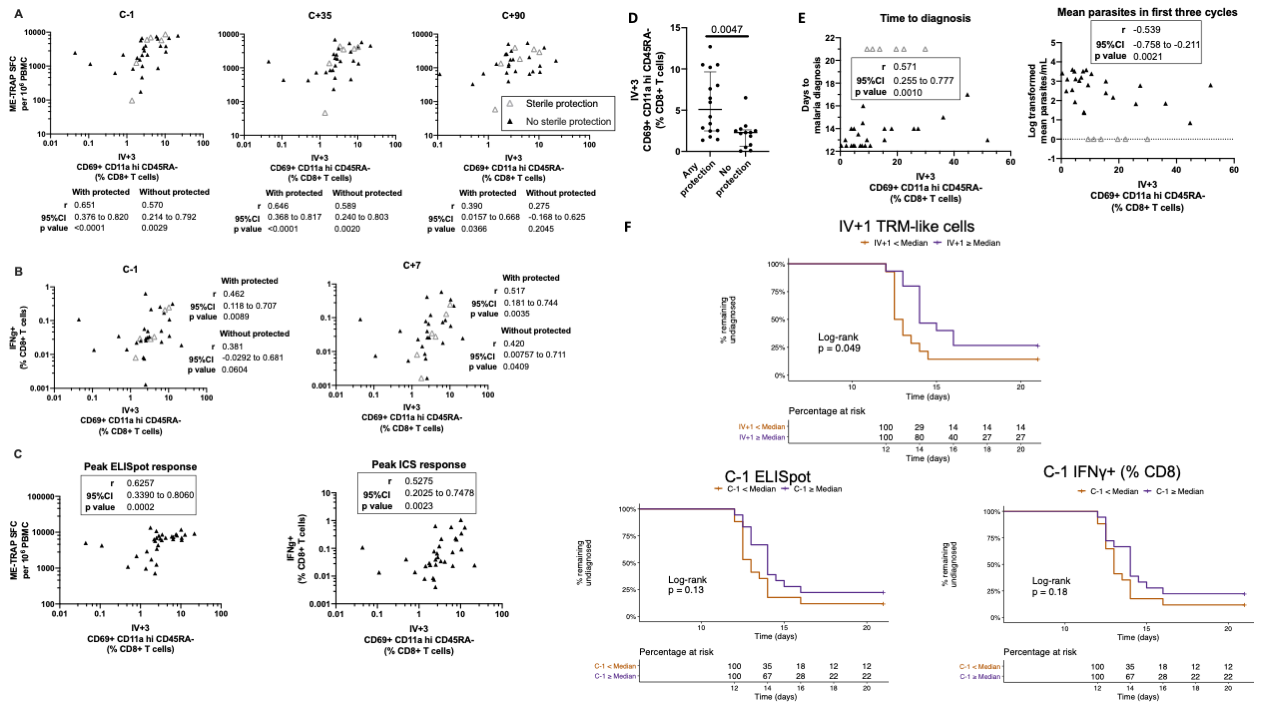

Supplement: Supplementary Figure 1 — Trial overview and timeline. Detailed schematic of trial. The final study cohort included 35 volunteers, across five groups, with group sizes for the first four (intervention) groups as indicated. Not shown in this figure is the infectivity control group (N=5) that did not receive any vaccinations and underwent CHMI with the vaccinated groups. All intervention group volunteers received either two or three vaccines of viral vectors encoding ME-TRAP. Doses were as indicated. Fine needle aspirates occurred between 16 and 24 days after IV vaccination/boost. Controlled Human Malaria Infection (CHMI, challenge) of volunteers by infected mosquitoes was carried out in a CL3 suite. Volunteers underwent CHMI on two separate days (4/2/2019 and 5/2/2019) and participants were equally and randomly divided across these days. Infectious mosquitos were provided by the Walter Reed Army Institute of Research. Volunteers were exposed to the bites of five infectious mosquitoes per participant for 5-10 minutes. Fed mosquitoes were individually dissected and assessed for sporozoite load, to ensure all fed mosquitoes were infected with P. falciparum. Volunteers were followed up twice per day from five days after challenge. Volunteers were treated with 20mg artemether/120mg lumefantrine (Riamet) upon malaria diagnosis or 21 days after challenge. A diagnosis of malaria infection was made given the following criteria: 1) The presence of symptoms suggestive of malaria in addition to a qPCR result indicating ≥ 1,000 parasites/mL OR 2) Lack of symptoms with a qPCR result indicating ≥ 10,000 parasites/mL. None of the volunteers in the infectivity control group achieved sterile protection. [file DataSheet_2.zip › Supplementary Figure 12.tiff]

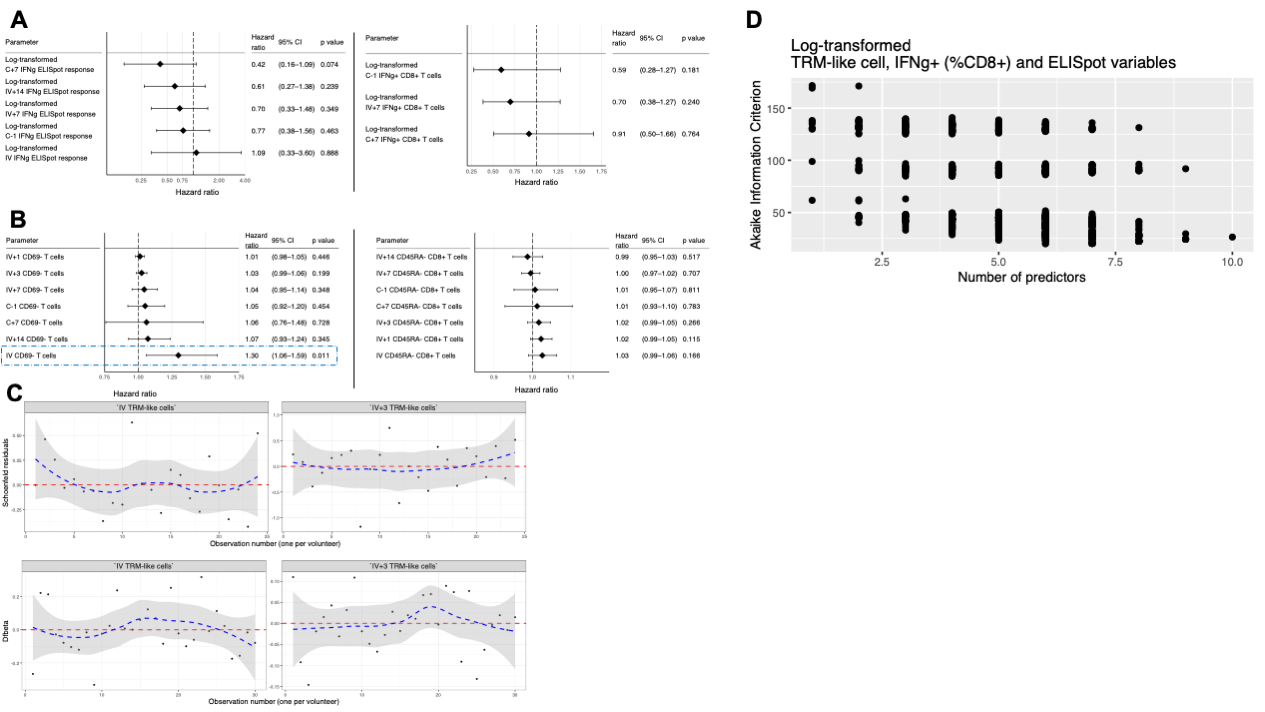

Supplement: Supplementary Figure 1 — Trial overview and timeline. Detailed schematic of trial. The final study cohort included 35 volunteers, across five groups, with group sizes for the first four (intervention) groups as indicated. Not shown in this figure is the infectivity control group (N=5) that did not receive any vaccinations and underwent CHMI with the vaccinated groups. All intervention group volunteers received either two or three vaccines of viral vectors encoding ME-TRAP. Doses were as indicated. Fine needle aspirates occurred between 16 and 24 days after IV vaccination/boost. Controlled Human Malaria Infection (CHMI, challenge) of volunteers by infected mosquitoes was carried out in a CL3 suite. Volunteers underwent CHMI on two separate days (4/2/2019 and 5/2/2019) and participants were equally and randomly divided across these days. Infectious mosquitos were provided by the Walter Reed Army Institute of Research. Volunteers were exposed to the bites of five infectious mosquitoes per participant for 5-10 minutes. Fed mosquitoes were individually dissected and assessed for sporozoite load, to ensure all fed mosquitoes were infected with P. falciparum. Volunteers were followed up twice per day from five days after challenge. Volunteers were treated with 20mg artemether/120mg lumefantrine (Riamet) upon malaria diagnosis or 21 days after challenge. A diagnosis of malaria infection was made given the following criteria: 1) The presence of symptoms suggestive of malaria in addition to a qPCR result indicating ≥ 1,000 parasites/mL OR 2) Lack of symptoms with a qPCR result indicating ≥ 10,000 parasites/mL. None of the volunteers in the infectivity control group achieved sterile protection. [file DataSheet_2.zip › Supplementary Figure 13.tiff]
