# Supplementary material for: Deep Immune Phenotyping and Single-Cell Transcriptomics Allow Identification of Circulating TRM-Like Cells Which Correlate With Liver-Stage Immunity and Vaccine-Induced Protection From Malaria
Source: Front Immunol. 2022 Feb 7;13:795463. doi: 10.3389/fimmu.2022.795463 (PMC8859435; doi:10.3389/fimmu.2022.795463)
Supplement: Supplementary file 3 [file DataSheet_3.docx]

# Antibody list

See tables 1 & 2 for lists of antibodies used in the study.

Table 1: Functional human antibodies

| Target | Clone | Isotype | Supplier | Cat. no. |
| --- | --- | --- | --- | --- |
| CD28 | CD28.2 | Mouse anti-human IgG1 κ | ThermoFisher | 16-0289-81 |
| CD49d | 9F10 | Mouse anti-human IgG1 κ | ThermoFisher | 16-0499-81 |

Table 2: Human antibodies for flow cytometry and intracellular cytokine staining

| Target | Fluorochrome | Clone | Isotype | Supplier | Cat. no. |
| --- | --- | --- | --- | --- | --- |
| Live-Dead Aqua | AmCyan | - | Anti-all species | ThermoFisher | L34957 |
| Ki67 | PE/Cy7 | Ki-67 | Mouse anti-human IgG1 κ | BioLegend | 350526 |
| IFN-γ | PE | 4S.B3 | Mouse anti-human IgG1 κ | BioLegend | 502509 |
| CD8α | APC | SK1 | Mouse anti-human IgG1 κ | BioLegend | 344722 |
| CD8 | APC | RPA-T4 | Mouse anti-human IgG1, κ | BioLegend | 344721 |
| CD8 | BUV395 | RPA-T8 | Mouse anti-human | BD Biosciences | 563795 |
| CD69 | AF488 | FN50 | Mouse anti-human IgG1 κ | BioLegend | 310916 |
| CD69 | FITC | FN50 | Mouse anti-human IgG1, κ | BioLegend | 310904 |
| CD69 | AF647 | FN50 | Mouse anti-human, IgG1 κ | BioLegend | 310918 |
| CD56 | BV570 | 5.1H11 | Mouse anti-human IgG1, κ | BioLegend | 362540 |
| CD56 | PE | 39D5 | Mouse anti-human, IgG1 κ | BioLegend | 355504 |
| CD45RA | APC/Fire750 | HI100 | Mouse anti-human IgG2b κ | BioLegend | 304152 |
| CD45 | BV780 | 2D1 | Mouse anti-human IgG1, κ | BioLegend | 368528 |
| CD4 | BV570 | RPA-T4 | Mouse anti-human IgG1 κ | BioLegend | 300534 |
| CD4 | PECy7 | OKT4 | Mouse anti-human IgG2b, κ | BioLegend | 317414 |
| CD4 | BUV496 | Sk3 | Mouse anti-human, IgG1 κ | BD Biosciences | 612936 |
| CD3 | Pacific Blue | UCHT1 | Mouse anti-human IgG1, κ | BioLegend | 300431 |
| CD3 | Pacific Blue | OKT3 | Mouse anti-human, IgG2a κ | BioLegend | 317314 |
| CD279 (PD-1) | BV650 | EH12.2H7 | Mouse anti-human IgG1 κ | BioLegend | 329950 |
| CD279 | BV711 | NAT-103 | Mouse anti-human IgG1, κ | BioLegend | 367428 |
| CD20 | BV570 | 2H7 | Mouse anti-human IgG2b κ | BioLegend | 302332 |
| CD20 | BV510 | 2H7 | Mouse anti-human IgG2b, κ | BioLegend | 302340 |
| CD186 (CXCR6) | PerCP-Cy5.5 | K041E5 | Mouse anti-human, IgG2a κ | BioLegend | 356010 |
| CD161 | BV605 | HP-3G10 | Mouse anti-human, IgG1 κ | BioLegend | 339916 |
| CD16 | BV650 | 3G8 | Mouse anti-human IgG1, κ | BioLegend | 302042 |
| CD16 | FITC | 3G8 | Mouse anti-human, IgG1 κ | BioLegend | 302006 |
| CD14 | PE | M5E2 | Mouse anti-human IgG2a, κ | BioLegend | 301806 |
| CD11c | BV650 | 3.9 | Mouse anti-human, IgG1 κ | BioLegend | 301638 |
| CD11b | A700 | ICRF44 | Mouse anti-human IgG1, κ | BioLegend | 301356 |
| CD11a | A700 | HI111 | Mouse anti-human IgG1 κ | BioLegend | 301228 |
| CD11a | PE-Cy5 | HI111 | Mouse anti-human, IgG1 κ | BioLegend | 301210 |
| CD107a | BV605 | H4A3 | Mouse anti-human IgG1 κ | BioLegend | 328634 |
| CD103 | BV711 | Ber-ACT8 | Mouse anti-human IgG1 κ | BioLegend | 350222 |
| CD103 | BV605 | Ber-ACT8 | Mouse anti-human IgG1, κ | BioLegend | 350218 |
| TCR α/β | PEDazzle594 | IP26 | Mouse anti-human, IgG1 κ | BioLegend | 306726 |

# RNA Sequencing

Smart-Seq2 libraries were generated following the established protocol [31]. cDNA underwent pre-amplification by PCR for 24 cycles and post-PCR clean-up was performed using a Biomek 4000 Automated Liquid Handler (Beckman Coulter). Nextera library preparation was performed using a Mosquito LV genomics instrument (SPT Labtech). Libraries were sequenced on an Illumina NextSeq with 75bp paired-end reads.

# Statistics, software and versions

Prism version 8 (GraphPad Software Inc.) and RStudio (base R version 3.6.2) were used for analyses. Significance testing of differences between group means (for normally distributed data) used the two-tailed Student's t-test, or medians used the two-tailed Mann‑Whitney U‑test (MW). Correlations were analysed using Spearman's rank correlation coefficient (r) for non-parametric data. An α (probability of type I error) of 0.05 was used, and p values below this were considered statistically significant. In the event of multiple comparisons, two multiple comparison correction methods were used. If there were between five and fifteen tested comparisons, a Kruskal–Wallis test (KW) with Dunn's correction for comparisons between multiple groups was applied. If there were more than fifteen tested comparisons, a two-stage linear step-up, Benjamini, Krieger and Yekutieli approach was used to control the false discovery rate. The false discovery rate was in most cases was set at 5%, if not lower. This approach determines a p value threshold, below which comparisons can be deemed true discoveries. Unadjusted p values were presented and those representing true discoveries were highlighted.

Immunological correlations with time to malaria diagnosis or other variables were pre-specified and prioritised analysis of T cell subsets based on observations from preclinical studies using prime-target vaccination. For log-transformed PCR data, an arbitrary value of 1 was added to each PCR value to allow log transformation of 0.0 values (y=log10(1+PCR value)). The mean number of parasites per replication cycle (area under the curve) was calculated using the trapezoid rule (5), included all parasite count values and was compared between groups. Replication cycles were defined by the date of CHMI: days 6.5‑8 (first cycle post‑hepatocyte release), days 8.5‑10 (second cycle) and days 10.5‑12 (third cycle) post-CHMI. Analyses compared median time to malaria diagnosis between strata dichotomised by median T cell frequency. Univariate and multiple regression Cox proportional hazards models were fitted using flow cytometry, ELISpot response and ICS data with T cell response as the independent variable and time to malaria diagnosis as the dependent variable. T cell populations which were associated with statistically significant reductions in hazard ratio were considered in the multiple regression analysis and, in addition, predictors with biologically relevant relationship to protection from malaria were considered in multiple regression. Models were fit using log-transformed data. Multiple regression model development was carried out in a forward stepwise manner: covariates were added one at a time, at each stage including the covariate which resulted in the model with the lowest Akaike Information Criterion (AIC) value. AIC was used as an aid for choosing between competing (nested) models. Lower AIC values indicated a preferred model. Specifically, the Δ𝑖 method was used, where Δ𝑖=AICi – AICmin (9). AICi was the AIC of the 𝑖th model, and AICmin was the lowest AIC obtained among the set of models examined (ie. the preferred model). As suggested by Burnham and Anderson (9), models with Δ𝑖 > 10 were considered as not descriptive of the dependent variable. Collinearity occurs when two or more independent variables are highly correlated with one another in a regression model. The variance inflation factor measures how well an independent variable is explained by other independent variables (48). The variance inflation factor was used to assess for collinearity.

# Bioinformatic analyses

T-distributed Stochastic Neighbour Embedding (tSNE) is a machine learning algorithm for nonlinear dimensionality reduction and visualisation. When run using FlowJo (Version 10, Becton, Dickinson and Company), the settings used included: 1000 iterations, perplexity 50, eta 200 and theta 0.5. tSNEs were run a number of times to ensure relative stability of inferences. In these iterations, the clusters were indifferent to variations in perplexity.

Trim Galore v0.6.5 ([github.com/FelixKrueger/TrimGalore](https://github.com/FelixKrueger/TrimGalore)) was used to trim sequencing adapters (-q 20 -m 15). Trimmed reads were aligned to the human genome (hg38 assembly) plus added ERCC “spike-in” sequences using STAR v2.5.3a [1]. Unstranded read counting was carried out and Ensembl gene counts were generated using featureCounts v1.6.0 [2]. Mini-bulk secondary QC considered absolute read counts, uniquely mapped reads and reads assigned to transcripts. Genes with more than 10 counts across all samples were kept for further analyses. Single cell secondary QC was performed on cells by considering total counts, total features, the proportion of mitochondrial DNA, cell cycle effects and mapping rate. Secondary QC was performed on samples by considering absolute read counts, uniquely mapped reads and reads assigned to transcripts. Two samples were removed due to low total read counts, and another four were removed due to low library complexity. Genes with more than ten counts across all samples were kept for further analyses.

DESeq2 v3.10 [3] was used for normalisation and feature selection in analysis of the mini-bulk experiment. The Wald test was the default used for hypothesis testing. Multiple test correction was applied by using the Benjamini and Hochberg/false discovery rate (FDR) method [4]. The binary logarithm of fold change (log_2_(FC)) values were shrunk using the Approximate Posterior Estimation for generalised linear models [5]. The differential gene expression (DGE) list for functional analyses was taken after shrinkage. A mix of over-representation analyses (ORA) and functional class scoring (FCS) were used. For ORA, the DGE list was composed of genes with an FDR<0.05 and hypergeometric tests were used to determine whether the input gene list was over-represented. The FCS performed was Gene Set Enrichment Analysis (GSEA) [6]. Multiple test correction was applied by using the FDR method. GSEA analyses used the log_2_(FC) values from the DGE list. The R packages clusterProfiler v3.14.3, fgsea v1.12, enrichplot v1.6 and pathview 1.26 were used to perform these analyses.

Single cell quality control (QC) was performed according to best practice[7, 8], and involved the total number of counts per cell (median ±3 median absolute deviations), the total number of features per cell (median ±3 median absolute deviations) and the percentage of mitochondrial DNA (<5%). Of the 736 cells commencing secondary QC, 629 remained for further analysis. Genes appearing in nine or fewer cells were removed prior to differential expression analysis. There were no apparent batch, plate or volunteer effects causing the removal of cells, nor was there a noticeable batch effect in the remaining cells.

Seurat v3.1.4 [9, 10] was used for normalisation, variance stabilisation and feature selection. The R package sctransform (via Seurat v3.1.1) was used to perform normalisation and variance stabilisation for PCA and initial clustering using the 2000 most variable features [11]. PCA was performed using Seurat, and Uniform Manifold Approximation and Projection (UMAP) [12, 13] used the first 12 principal components (PC) from the PCA. Differential expression of data normalised with LogNormalised method was based on the non-parameteric Wilcoxon rank sum test. P values were adjusted using Bonferroni correction based on the total number of genes in the dataset and only adjusted p values (padj) were used. DGE was designated as genes with an absolute value of the natural logarithm of the fold change (ln(FC))>0.5 and padj<0.05, unless otherwise indicated.

Pseudotime analysis was performed with slingshot v1.4.0 (33). Slingshot uses clusters of cells to uncover global structure by interpreting Euclidean distances. The slingshot package then converts this structure into smooth lineages represented by a one-dimensional variable, called "pseudotime." Single Cell Signature Explorer was used for scoring of single cells according to multi-gene features (34). Both pseudotime and signature analyses were projected onto the UMAP. TraCeR was used to reconstruct single-cell α and β T cell receptors (TCR) and complementarity determining regions (CDR) (35). Prior to running TraCeR 46 cells were removed due to low read counts (identified by small file size (<2Mb), incompatible with adequate sequencing). Therefore TraCeR was run on 690 cells in two batches. TraCeR Assemble and Summarise functions were implemented using Bowtie2 v2.4.2 (36, 37), Trinity v2.11.0 (38), IgBLAST v1.15.0 (39) and Kallisto v0.46.1 (using the --small_index argument and providing the GRCh38.p13 assembly) (40). MAIT cells were defined by TCR, as any cells that productively expressed *TRAV1-2* paired with i) *TRAJ33*, ii) *TRAJ12* or iii) *TRAJ20*, as previously described (41).

# Ex-vivo interferon-γ (IFNg) Enzyme-Linked Immunosorbent Spot (ELISpot)

*Ex vivo* (18-hour stimulation) ELISpot assays were performed at several time points as previously described [15, 16]. Ex vivo (18 to 20-hour stimulation) ELISpot assays were performed using Multiscreen IP ELISpot plates (Millipore), human IFNγ SA-ALP antibody kits (Mabtech) and BCIP NBT-plus chromogenic substrate (Moss Inc.), using freshly collected cells. The coating antibody was monoclonal anti-human IFNγ 1-D1K (mouse IgG1), and the detection antibody was biotinylated monoclonal 7-B6-1 (mouse IgG1). Cells were cultured in R10, with FCS previously screened for low reactivity. All samples were stimulated with a pool of TRAP peptides from two species of Plasmodium falciparum or with R10 medium (as a negative/unstimulated control). PBMC ELISpots were plated with 100,000 lymphocytes per well, in triplicate. FNA ELISpots were plated with 50,000 lymphocytes per well, in duplicate. Spots were counted using an ELISpot counter (Autoimmun Diagnostika, Germany). Results are expressed as the mean of the replicate IFNγ spot-forming colonies (SFC) per million PBMC. Background responses in un-stimulated control wells were less than 15 spots and were subtracted from those measured in peptide-stimulated wells.

References

1. Dobin A, Davis CA, Schlesinger F, Drenkow J, Zaleski C, Jha S, et al. STAR: ultrafast universal RNA-seq aligner. Bioinformatics. 2013;29(1):15-21.

2. Liao Y, Smyth GK, Shi W. featureCounts: an efficient general purpose program for assigning sequence reads to genomic features. Bioinformatics. 2014;30(7):923-30.

3. Love MI, Huber W, Anders S. Moderated estimation of fold change and dispersion for RNA-seq data with DESeq2. Genome Biol. 2014;15(12):550.

4. Benjamini Y, Hochberg Y. Controlling the False Discovery Rate: A Practical and Powerful Approach to Multiple Testing. Journal of the Royal Statistical Society Series B (Methodological). 1995;57(1):289-300.

5. Zhu A, Ibrahim JG, Love MI. Heavy-tailed prior distributions for sequence count data: removing the noise and preserving large differences. Bioinformatics. 2019;35(12):2084-92.

6. Mootha VK, Lindgren CM, Eriksson KF, Subramanian A, Sihag S, Lehar J, et al. PGC-1alpha-responsive genes involved in oxidative phosphorylation are coordinately downregulated in human diabetes. Nat Genet. 2003;34(3):267-73.

7. Amezquita RA, Lun ATL, Becht E, Carey VJ, Carpp LN, Geistlinger L, et al. Orchestrating single-cell analysis with Bioconductor. Nat Methods. 2020;17(2):137-45.

8. Luecken MD, Theis FJ. Current best practices in single-cell RNA-seq analysis: a tutorial. Mol Syst Biol. 2019;15(6):e8746.

9. Butler A, Hoffman P, Smibert P, Papalexi E, Satija R. Integrating single-cell transcriptomic data across different conditions, technologies, and species. Nat Biotechnol. 2018;36(5):411-20.

10. Stuart T, Butler A, Hoffman P, Hafemeister C, Papalexi E, Mauck WM, 3rd, et al. Comprehensive Integration of Single-Cell Data. Cell. 2019;177(7):1888-902 e21.

11. Hafemeister C, Satija R. Normalization and variance stabilization of single-cell RNA-seq data using regularized negative binomial regression. Genome Biol. 2019;20(1):296.

12. Becht E, McInnes L, Healy J, Dutertre CA, Kwok IWH, Ng LG, et al. Dimensionality reduction for visualizing single-cell data using UMAP. Nat Biotechnol. 2018.

13. McInnes et al., (2018). UMAP: Uniform Manifold Approximation and Projection. Journal of Open Source Software, 3(29), 861.

14. Street K, Risso D, Fletcher RB, Das D, Ngai J, Yosef N, et al. Slingshot: cell lineage and pseudotime inference for single-cell transcriptomics. BMC Genomics. 2018;19(1):477.

15. Hodgson SH, Ewer KJ, Bliss CM, Edwards NJ, Rampling T, Anagnostou NA, et al. Evaluation of the efficacy of ChAd63-MVA vectored vaccines expressing circumsporozoite protein and ME-TRAP against controlled human malaria infection in malaria-naive individuals. J Infect Dis. 2015;211(7):1076-86.

16. Ogwang C, Afolabi M, Kimani D, Jagne YJ, Sheehy SH, Bliss CM, et al. Safety and immunogenicity of heterologous prime-boost immunisation with Plasmodium falciparum malaria candidate vaccines, ChAd63 ME-TRAP and MVA ME-TRAP, in healthy Gambian and Kenyan adults. PLoS One. 2013;8(3):e57726.
